# Metal‐Organic Framework‐Based Micro‐/Nanomotors for Wastewater Remediation

**DOI:** 10.1002/smsc.202400110

**Published:** 2024-06-26

**Authors:** Karim El‐Naggar, Yangyang Yang, Wenjie Tian, Huayang Zhang, Hongqi Sun, Shaobin Wang

**Affiliations:** ^1^ School of Chemical Engineering The University of Adelaide North Terrace Adelaide SA 5005 Australia; ^2^ Department of Chemistry Faculty of Science Ain Shams University Abbassia Cairo 11566 Egypt; ^3^ Institute of Green Chemistry and Chemical Technology School of Chemistry & Chemical Engineering Jiangsu University Zhenjiang 212013 China; ^4^ School of Molecular Sciences Faculty of Science The University of Western Australia Perth WA 6009 Australia

**Keywords:** adsorptions, degradations, micro‐/nanomotors, metal‐organic frameworks, sensing, wastewater remediation

## Abstract

Micro‐/nanomotors (MNMs) in water remediation have garnered significant attention over the past two decades. More recently, metal‐organic framework‐based micro‐/nanomotors (MOF‐MNMs) have been applied for environmental remediation; however, a comprehensive summary of research in this research area is yet to be reported. Herein, a review is presented to cover the recent advances in MOF‐MNMs and their various applications in wastewater remediation. The review presents a comprehensive introduction to MNMs, including different propulsion approaches, fabrication, and functionalization strategies, in addition to the unique features of MOF‐MNMs. The conception and various synthetic routes of MOF‐MNMs are extensively covered and the implementation of MOF‐MNMs in water‐related applications, including adsorption, degradation, sensing, and disinfection of different pollutants, is in depth discussed. Meanwhile, the propulsion and mechanism of action behind each MOF‐MNM are systematically studied. Finally, the review provides insights into the challenges and perspectives to build more effective MOF‐MNMs to cover versatile applications for wastewater treatment.

## Graphical abstract

This review explores recent advancements in metal‐organic framework‐based micro/nanomotors (MOF‐MNMs) for wastewater remediation. It comprehensively covers their propulsion mechanisms, fabrication, functionalization, and unique features, highlighting their applications in adsorption, degradation, sensing, and disinfection of pollutants. Additionally, it discusses challenges and future perspectives to enhance MOF‐MNMs’ effectiveness in water treatment.

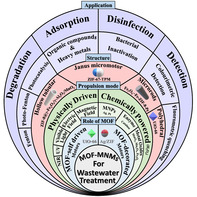

## 1 Introduction

Due to rapid industrialization, careless human activities, as well as the current COVID‐19‐related pandemic, water is widely polluted by different types of contaminants, including toxic metals,^[^ ^1^ ^]^ organic compounds,^[^ ^2^ ^]^ more emerging contaminants (personal care, agricultural, and pharmaceutical products, as well as microplastics),^[^ ^3^ ^]^ and pathogens.^[^ ^4^ ^]^ All these types of pollutants are highly toxic and can cause detrimental effects on not only human health but also all other living creatures, in both short‐ and long‐term exposure.

Currently, researchers have intensively used different techniques for water/wastewater treatment, ranging from adsorption,^[^ ^5^ ^]^ advanced oxidation processes,[^2^, ^6^] membrane filtration,^[^ ^7^ ^]^ electrochemical oxidation,^[^ ^8^ ^]^ to microwave‐assisted catalytic oxidation.^[^ ^9^ ^]^ Over the past two decades, micro‐/nanomotors (denoted as MNMs) have gained much research attention in different areas. MNMs are a new type of nanomaterials that can convert one form of energy into motion. Because of their self‐propulsion capabilities, MNMs have been intensively used in different applications (e.g., biomedical applications, environmental remediation, etc.).

For example, Yang et al. applied the flask‐like FeO_*x*_@MnO_2_@SiO_2_ micromotor for the catalytic degradation of antibiotics (Naproxen) in an H_2_O_2_/peroxymonosulfate system.^[^ ^10^ ^]^ Ho and Yoo reported a manganese dioxide‐based motor for organic dye removal.^[^ ^11^ ^]^ Shang and co‐workers investigated the removal of heavy metal ions using self‐propelled structural color cylindrical micromotors.^[^ ^12^ ^]^ Pumera et al. tested the fungicidal activity of single‐component BiVO_4_ micromotor under visible light irradiation.^[^ ^13^ ^]^ MNMs have been also applied in biological applications. For instance, Wang et al. reported the doxorubicin drug delivery inside the stomach by a self‐propelled poly (aspartic acid)/iron‐zinc microrocket.^[^ ^14^ ^]^ In addition, Pumera and his group implemented the concept of microsurgery to remove cancer cells using magnetically driven Au/Ag/Ni microrobotic scalpels.^[^ ^15^ ^]^

Due to the widespread use of MNMs, various micro/nanomaterials (generally made from inorganic, organic, organometallic, biological, and polymeric materials as well as their composites) have been fabricated.^[^ ^13^, ^16^ ^]^ Among them, TiO_2_ (Au/B‐TiO_2_), metals/silica (Au/SiO_2_, Pt/SiO_2_), mixed metals (Mg/Ag, Au/Pt, and Ni/Ag), activated carbon, graphene oxide (GO), reduced GO (rGO) and its composites (rGO–SiO_2_–Pt Janus micromotors), polymers (PEG/PS), carbon‐based materials (CNTs/Pt and Au‐modified WO_3_@C), and other metal‐based materials (Fe_2_O_3_, Fe_3_O_4_, Cu_2_O/Au, BiOI/Au, Ag_3_PO_4_, MnO_2_/Ag, WS_2_, WO_3_/Ag, CaCO_3_, Pt‐ZnIn_2_S_4_, Co_3_O_4_/CeO_2_, ZnO‐Au, PbS QDs, and MnFe_2_O_4_) were studied in detail.^[^ ^17^ ^]^

Metal‐organic framework (MOF) was first introduced in 1995 by Yaghi.^[^ ^18^ ^]^ MOFs are defined as a class of porous organic‐inorganic hybrids via the assembly of both metal ions, known as secondary building units (SBUs), and organic ligands or linkers, located between the SBUs to give crystalline structures with unique porous textures. Currently, more than 20 000 different MOFs have been prepared, and this number is still increasing.^[^ ^18^ ^]^ Because of their high specific surface areas of up to 6000–10 000 m^2^ g^−1^, facile tunability of pore size and shape, and extraordinary chemical, thermal, and water stability, MOFs have been used in a wide range of applications,^[^ ^19^ ^]^ including gas adsorption and storage,^[^ ^20^ ^]^ separation,^[^ ^21^ ^]^ biomedical processes (e.g., drug delivery),^[^ ^22^ ^]^ water treatment (e.g., adsorption and photocatalytic degradation of organic pollutants, photoreduction of toxic metals),[^7^, ^23^] and analytical sensing.^[^ ^24^ ^]^

**Figure 1:**
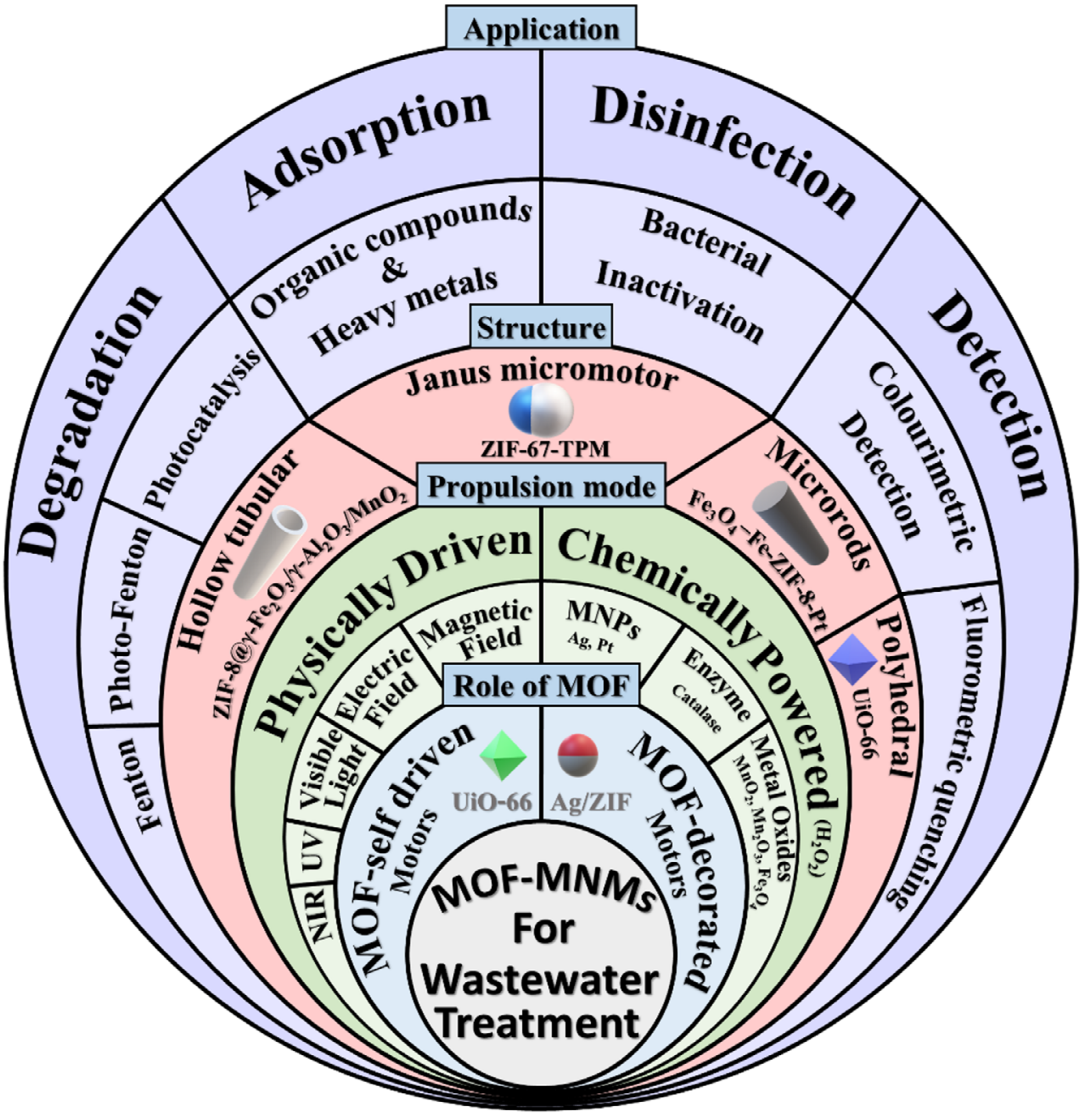
Schematic illustration highlighting the different aspects of MOF‐MNMs reported for wastewater remediation (role of MOF in MOF‐MNMs, propulsion mode, different structures, and applications).

Integrating the superior characteristics of MOFs with the autonomous movement of the micro/nanomotors can produce MOF‐based micro/nanomotors (abbreviated MOF‐MNMs) and offer a new paradigm for different applications, especially wastewater remediation (**Figure** **1**). More importantly, MOF‐MNMs can be powered by different energy sources, including bubble propulsion,^[^ ^25^ ^]^ light,^[^ ^26^ ^]^ near‐infrared (NIR) irradiation,^[^ ^27^ ^]^ magnetic field,^[^ ^28^ ^]^ and electric field,^[^ ^29^ ^]^ in addition to the self‐fuelled MOF.^[^ ^30^ ^]^

MNMs have been covered from different aspects by a wide list of review articles. For example, Ye et al. summarized and discussed the design and fabrication of MNMs for environmental applications.^[^ ^31^ ^]^ Zha et al. gave a comprehensive overview of tubular micromotors, from propulsion to applications.^[^ ^32^ ^]^ Pumera et al. summarized the different fabrication techniques for micro‐/nanoscale motors designed for different applications.^[^ ^33^ ^]^ Chang et al. published a review about nature‐inspired MNMs covering and providing an overview of the various strategies to build motors that mimic the morphology, structure, function, or propulsion of living things.^[^ ^34^ ^]^ Wong et al. classified the fabrication of MNMs based on the materials used in synthesis, such as inorganic, organometallic, and polymer motors.^[16c]^ Lin et al. summarized the different strategies to integrate diverse functional components into different architectures of microscale motors.^[^ ^35^ ^]^ Zhang et al. reported the various functionalization techniques of MNMs, including chemical, biological, and self‐functionalization strategies.^[^ ^36^ ^]^ Yang et al. summarized the different mechanisms to control the propulsion of both synthetic MNMs and biohybrid motors.^[^ ^37^ ^]^ In addition, Tu et al. discussed the recent advancements in motion manipulation of MNMs by different approaches.^[^ ^38^ ^]^ For the source of energy used with MNMs, Sánchez et al. summarized the various aspects of chemically powered MNMs,^[^ ^39^ ^]^ whereas Xu et al. reviewed the fuel‐free synthetic MNMs powered using external physical stimulus.^[17o]^

Wang et al. provided an in‐depth summary of the various biomedical applications of MNMs.^[^ ^40^ ^]^ Moreover, Tezel et al. focused in their review on the role of MNMs in drug delivery.^[^ ^41^ ^]^ Wang et al. overviewed the implementation of MNMs in cell transportation and microsurgery.^[^ ^40^ ^]^ Various reviews have discussed the application of MNMs in environmental remediation, especially water purification.[^16^, ^17^, ^42^] Besides, MOF‐MNMs have been covered by different perspectives and reviews. Liu et al. highlighted the recent success of biocatalytic MOF‐based motors in healthcare and water treatment.^[^ ^43^ ^]^ Pumera et al. provided an overview of the synthesis and different propulsion mechanisms.^[^ ^44^ ^]^ Vikrant and Kim introduced a perspective on applying MOF‐MNMs in environmental applications, such as adsorptive and catalytic removal of pollutants.^[^ ^45^ ^]^ Falahati et al. examined the application of MOF‐MNMs in the targeted treatment of cancer.^[^ ^46^ ^]^ Escarpa et al. reviewed the implementation of MOF‐MNMs in biomedical applications, analytical sensing, and environmental remediation.^[^ ^47^ ^]^ Yang et al. discussed biomedical applications, including chemotherapy and antibacterial therapy, in addition to the applications in the environmental field.^[^ ^48^ ^]^ Despite these numerous works about MOF as MNMs, to our knowledge, there is no review focusing on MOF‐MNMs for wastewater remediation.

Consequently, this review focuses on the current status of MOF‐MNMs and their applications in wastewater remediation (Figure 1). The review began with a comprehensive introduction to the fundamental aspects of MNMs, discussing the different energy sources, fabrication, and functionalization methods. The conception and different synthetic approaches of MOF‐MNMs were explored. In addition, the implementation of MOF‐MNMs in water‐related applications, including adsorption, degradation, sensing, and disinfection of different pollutants, was then discussed in depth. The synthesis, propulsion, and mechanism of action behind each MOF‐MNM were systematically studied. Finally, we provided some research perspectives to build efficient MOF‐MNMs to remove different kinds of water pollutants.

## 2 Micro‐/Nanomotors (MNMs)

### 2.1 Fabrication of MNMs

MNMs (engines, swimmers, machines, or robots) are a new generation of synthesized micro‐/nanoscale materials with engineered autonomous or self‐movement abilities. These materials could move and interact with the surrounding environment autonomously, accomplishing a desired action, or a group of actions based on their components and designs.^[^ ^49^ ^]^ As a result of the different criteria related to the MNMs’ structures, like the material nature, dimension, as well as architectures, numerous fabrication or engineering methods have been extensively used.^[^ ^33^, ^39^ ^]^ Most of these strategies involve preparing the motor framework, which determines the overall motor properties, such as structure, mode of action, motion mechanism, and speed.^[^ ^33^ ^]^

**Figure 2:**
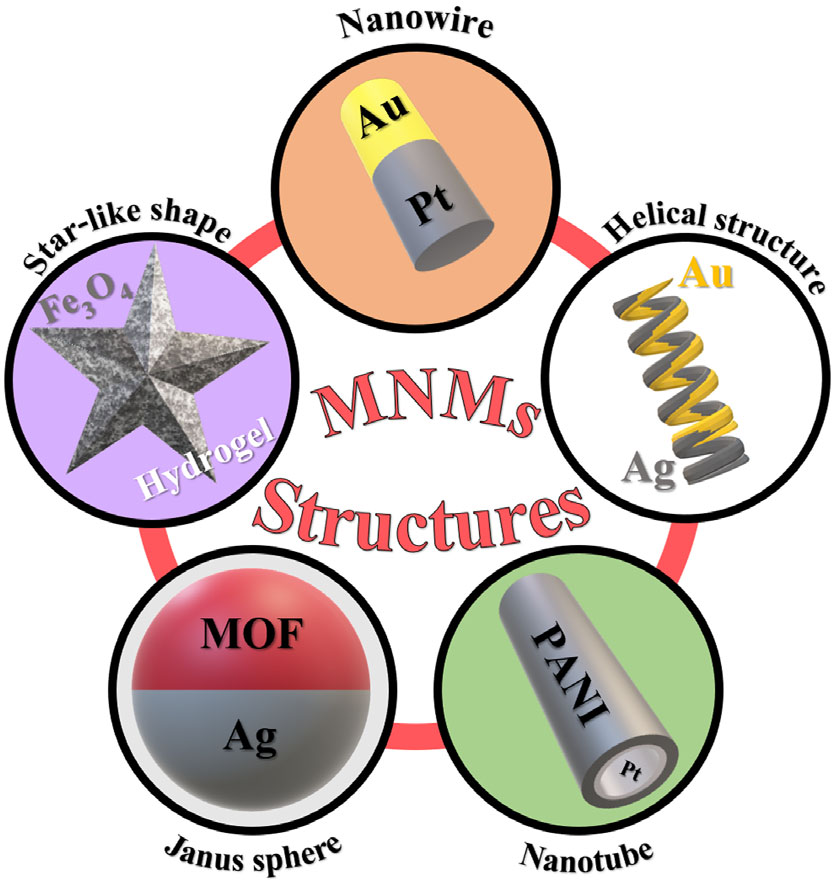
The different architectures of MNMs.

Electrochemical/electroless deposition (membrane template‐assisted electrodeposition, electrochemical and electroless deposition based on other templates, and asymmetric bipolar electrodeposition),^[^ ^50^ ^]^ physical vapor deposition (conventional physical vapor deposition and glancing angle deposition),^[^ ^51^ ^]^ strain engineering (rolled‐up technology for micro‐/nanotubes and self‐scrolling technique for helical micromotors),^[^ ^52^ ^]^ 3D direct laser writing,^[^ ^53^ ^]^ assembly of materials (layer‐by‐layer assembly, assembly and encapsulation of micro/nanoparticles (NPs), and assembly and incorporation of synthetic molecules)^[^ ^54^ ^]^ are common techniques for the fabrication of MNMs. Currently, MNMs can be engineered with a diversity of architectures,[^16^, ^17^, ^31^, ^32^, ^35^, ^37^, ^39^, ^55^] as illustrated in **Figure** **2**, including rods/wires, tubes, Janus sphere, helices, and twisted star‐like shape.^[^ ^56^ ^]^

**Figure 3:**
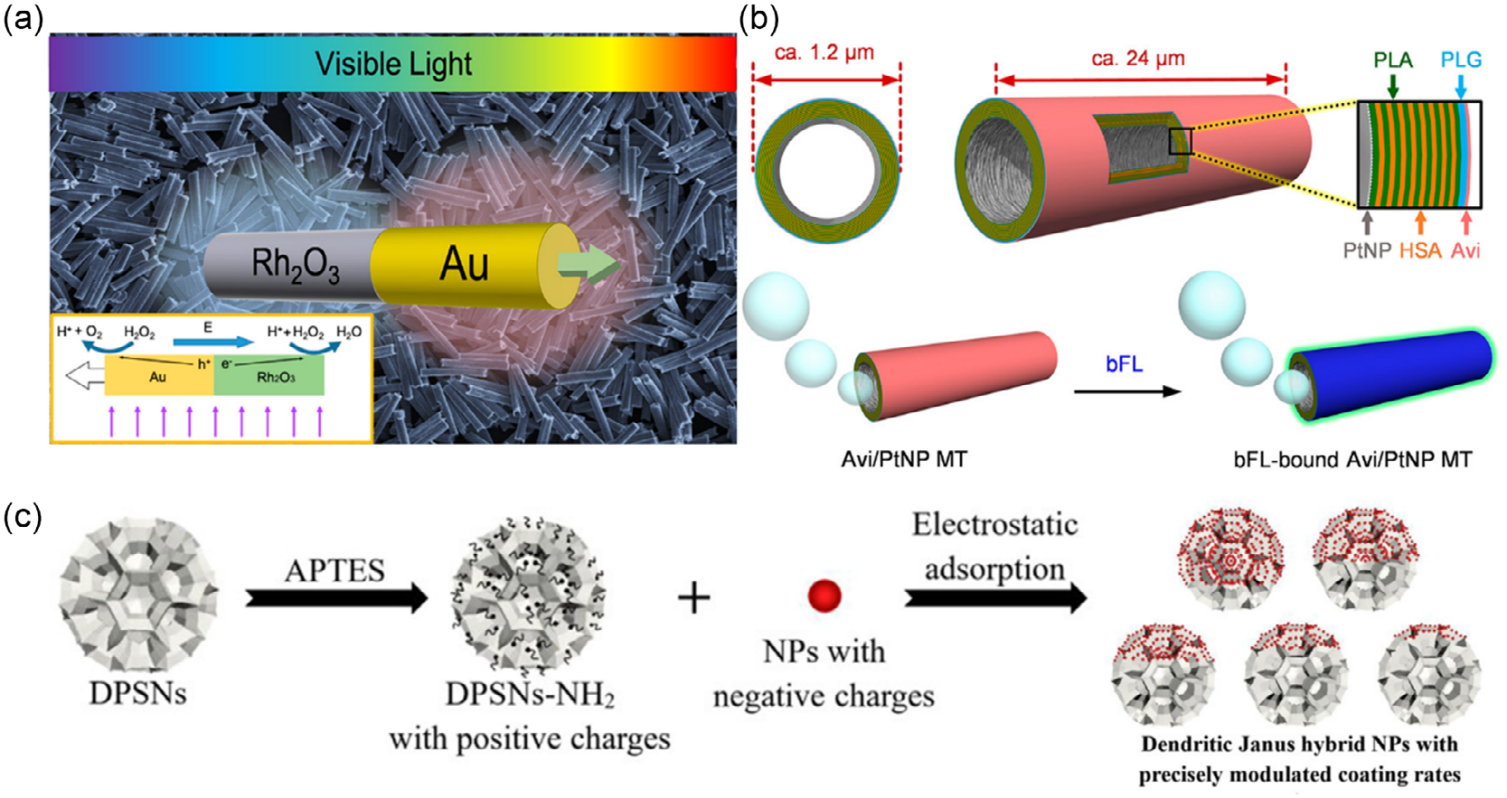
a) Structure and propulsion of Au–Rh_2_O_3_ nanorod (the inset shows the photocatalytic behavior and the corresponding chemical reaction occurred under illumination). Adapted with permission.^[17m]^ Copyright 2022, American Chemical Society. b) Schematic illustration of the protein microtube and adsorption behavior (PtNP: platinum NPs, HSA: human serum albumin, Avi: avidin, PLA: poly(L‐arginine) hydrochloride, PLG: poly(L‐glutamic acid), and bFL: pentylthioureidyl ‐fluorescein). Adapted with permission.^[^ ^57^ ^]^ Copyright 2019, American Chemical Society. c) Schematic illustration of the controllable fabrication of DPSN‐based Janus NPs. Adapted with permission.^[56e]^ Copyright 2019, American Chemical Society.

For instance, Cui et al. synthesized a nanorod motor of Au‐Rh_2_O_3_ that could be propelled in H_2_O_2_ fuel under the illumination of light from UV to the entire visible light spectrum (400–700 nm).^[17m]^ The motor contained a p–n heterojunction, where Rh_2_O_3_ behaved as a p‐type semiconductor and Au acted as an n‐type semiconductor. This junction enhanced the photocatalytic behavior, allowing the rods to move toward Au side via self‐electrophoteseis (**Figure** **3**a). Kamatsu et al. reported a self‐propelled protein microtube with a Pt NP‐coated internal wall.^[^ ^57^ ^]^ The tubes were propelled by the jetting oxygen bubbles from the open‐end terminus, which were catalytically generated from the decomposition of H_2_O_2_ over Pt NPs. However, the exterior surface of the tube was coated by avidin, to capture various biotinylated substances (Figure 3b). For Janus micromotors, Zhang et al. synthesized one based on dendritic porous silica NPs (DPSNs) precisely coated with different concentrations of Pt NPs.^[56e]^ The charge of the DPSNs was first modulated by introducing the aminopropyl triethoxy silane moiety, which gave positively charged DPSNs‐NH_2_ particles. The positive charges on the DPSNs‐NH_2_ particles could electrostatically attract the negatively charged NPs, and enable controlled covering of the surface (Figure 3c). The porous structure along with the precise modulation of coverages paves the way to understand their performance on cargo delivery. Thus, depending on the application, constituents of the motor, and the propulsion mode, it is easy to properly choose the suitable shape of the motor.

### 2.2 Functionalization and Structure Considerations of MNMs

The use of a pristine framework as MNMs can lead to nonspecific actions. Therefore, various surface modification and functionalization strategies have been developed and intensively used to enhance both the directionality and specificity of the MNMs to perform diverse and demanding tasks in different applications.^[^ ^33^, ^36^ ^]^ In other words, precise functionalization selection is essential for designing an effective MNM to match the intended application.^[^ ^33^ ^]^ Among these strategies, both chemical and biological functionalizations are considered the most used processes to cover most applications.

#### 2.2.1 Chemical Functionalization

**Figure 4:**
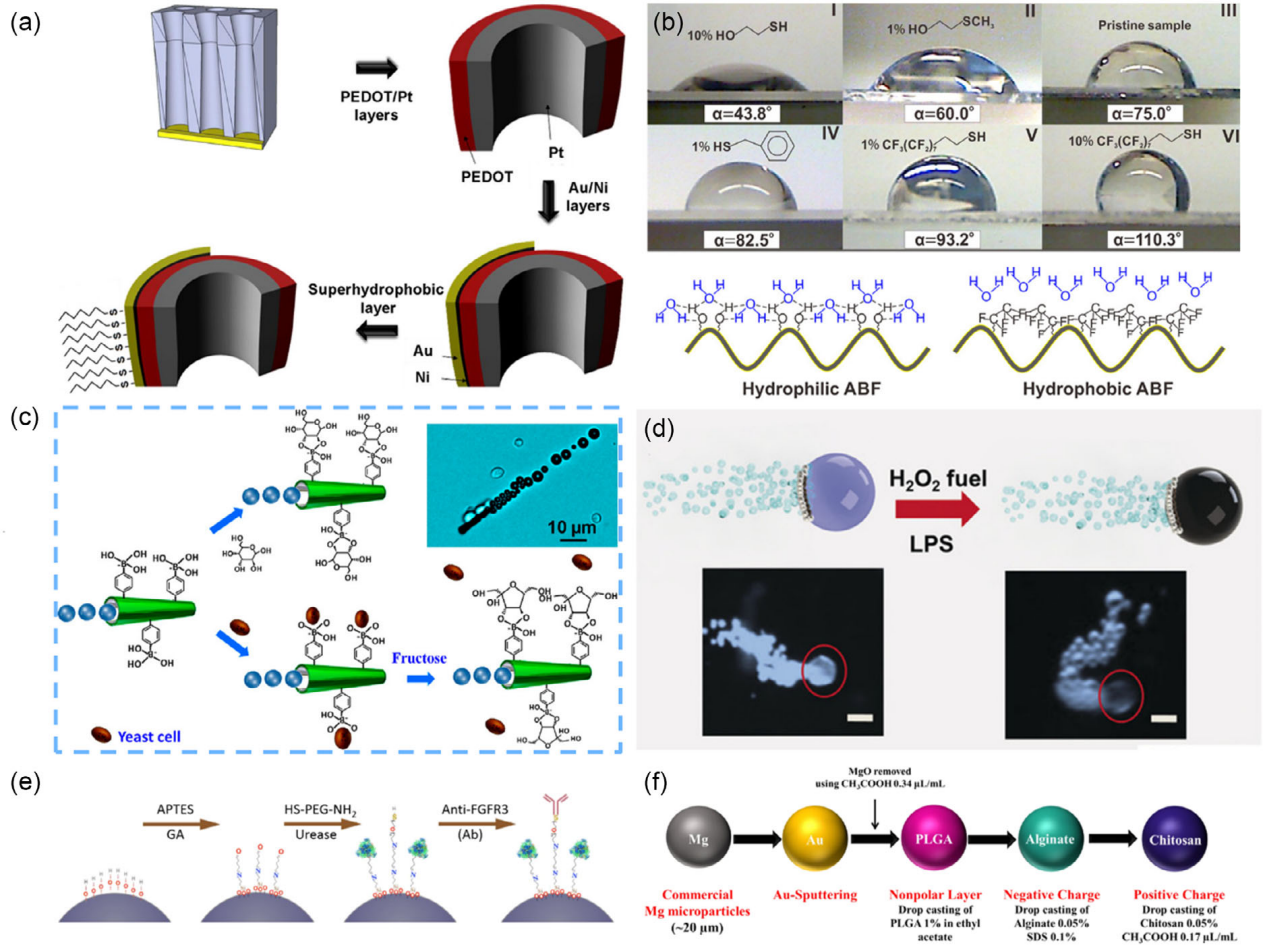
a) Fabrication and modification of the SAM‐Au/Ni/PEDOT/Pt micromotors. Adapted with permission.^[^ ^59^ ^]^ Copyright 2012, American Chemical Society. b) Water contact angles of different thiol‐ or thioether‐modified motors, and the interaction between water and microswimmers. Adapted with permission.^[^ ^61^ ^]^ Copyright 2018, American Chemical Society. c) The recognition of boronic acid‐modified motor with sugar and cells. Adapted with permission.^[58e]^ Copyright 2012, American Chemical Society. d) Bubble propulsion GQDs‐based motor before and after the interaction LPSs. Adapted with permission.^[^ ^63^ ^]^ Copyright 2017, John Wiley and Sons. e) Scheme illustrating the linking between (FGFR3)‐antibody and the PEG‐modified motor. Adapted with permission.^[^ ^70^ ^]^ Copyright 2019, American Chemical Society. f) Fabrication of the Mg/Au/PLGA/Alg/Chi Janus micromotors. Adapted with permission.^[^ ^68^ ^]^ Copyright 2017, The Royal Society of Chemistry.

Chemical modification is one of the key strategies employed to enhance the directionality and functionality of MNMs. It involves using organic and inorganic materials as surface capping agents, such as thiols, boronic acid and its derivatives, silanes, and NPs (such as Ag, Au, and carbon dots).[^17^, ^58^] For instance, a hydrostable micro‐/nanomaterial, when functionalized with a self‐assembled monolayer of hydrophobic compounds, could form a superhydrophobic MNM for oil removal from water. In this context, Guix et al. functionalized Au/Ni/PEDOT/Pt micromotor (where PEDOT stands for poly(3,4‐ethylenedioxythiophene)) with self‐assembled monolayers (SAMs) of different alkanethiols (with C6–C18 chain length) to give SAM‐Au/Ni/PEDOT/Pt, as illustrated in **Figure** **4**a.^[^ ^59^ ^]^ Alkanethiols are easily linked to the motor surface due to the strong interaction between sulfur and Au. The long alkane chain converted the motor into a superhydrophobic particle to be able to interact, collect, and transport oil droplets. Later, the same group utilized the good gold–thiol interaction and prepared a SAM‐modified hydrophobic Mg micromotor.^[^ ^60^ ^]^ This hydrophobic H_2_ bubble‐propelled motor showed promising activity to remove oil droplets from seawater, holding great promise for potential applications in a large area.

Moreover, Nelson et al. studied the effect of functionalization on the hydrophobicity and hydrophilicity of magnetic helical microswimmers.^[^ ^61^ ^]^ They used five different thiols and thioether‐based compounds terminated with different moieties, including hydroxyl‐, benzyl‐, and fluorine‐containing groups, to modify the surface of the motor.^[^ ^61^ ^]^ It was concluded that the microswimmer modified with compounds containing hydroxyl groups showed the lowest contact angles, making it hydrophilic in nature. Conversely, the swimmer with trifluoromethyl moieties showed the highest hydrophobicity (Figure 4b).^[^ ^61^ ^]^ Inspired by the well‐known molecular recognition of saccharides by boronic acid,^[^ ^62^ ^]^ Kuralay et al. functionalized a Ni/Pt microtube engine with a poly(3‐aminophenylboronic acid) for monosaccharide recognition.^[58e]^ The motor showed a high carbohydrate sensitivity, resulting in both capturing and transporting of glucose and binding and releasing yeast cells containing sugar moieties on their wall (Figure 4c). In addition, coupling graphene quantum dots (GQDs), a well‐known highly fluorescent NPs, with a micromotor could produce fluorescent MNMs. For instance, Escarpa et al. combined both phenylboronic acid (PABA) and GQDs to synthesize a highly fluorescent PABA‐GQDs/Fe_3_O_4_/Pt micromotor as an ultrafast sensor for lipopolysaccharides (LPSs) of the outer membrane of Gram‐negative bacteria.^[^ ^63^ ^]^ PABA acted as a highly specific recognition receptor for LPS, accompanied by fluorescence quenching to the GQDs (Figure 4d).

#### 2.2.2 Biological Functionalization

The chemical modification is unsuitable for in vivo applications, because of their biological toxicity and nonbiodegradability.^[^ ^64^ ^]^ Therefore, biological functionalization is considered the best alternative when targeting physiological environments without any detrimental effect. Biological functionalization uses synthetic and natural polymers, biomaterials, and functional biomolecules, with specific targeting abilities that can effectively and safely perform in vivo applications.^[^ ^64^, ^65^ ^]^

Chitosan (Chi), a positively charged natural polymer, has been reported to have strong adhesion properties to mucosal surfaces.^[^ ^66^ ^]^ Wei et al. synthesized a chitosan‐coated Mg‐TiO_2_‐Tox@RBC (Tox@RBC: endotoxin drug inserted in red blood cell as antigenic material) micromotor for drug delivery.^[^ ^67^ ^]^ After oral injection, the motor travelled through the gastrointestinal tract, where the mucosal adhesion of Chi enabled the active delivery of the antigens. In addition, Delezuk et al. synthesized a chitosan‐coated Mg micromotor with antibacterial activity.^[^ ^68^ ^]^ The electrostatic interaction between Chi and *Escherichia coli (E‐coli)* bacteria caused structural change and membrane deformation, followed by bacteria death. Other Chi derivatives, such as carboxymethyl, quaternized, and trimethyl chitosan, have been widely applied in biomedicine, which make them good candidates to be used in MNMs for biomedical applications.^[^ ^64^ ^]^ Alginate (Alg), another natural polymer, has been used to functionalize MNMs.^[^ ^64^ ^]^ Due to its negatively charged surface, it can be used to electrostatically adsorb positive substances.^[^ ^68^ ^]^ Alg‐hydrogel offers a 3D porous microstructure to protect the motor during propulsion.^[^ ^69^ ^]^

Synthetic polymers, such as polyethylene glycol (PEG) and polylactic*‐co*‐glycolic acid (PLGA), have been widely reported for functionalizing MNMs for biomedical applications. For instance, Sánchez et al. used PEG to prepare a urease‐powered nanomotor to target bladder cancer.^[^ ^70^ ^]^ PEG is used as a spacer to prevent the aggregation of the motor particles by providing steric hindrance between them. In addition, the linkage between PEG and the cysteine moieties in the antifibroblast growth factor receptor (FGFR3)‐antibody (Figure 4e) provided more specificity to the bladder cancer cells. Mixing more than one type of polymer could be beneficial in designing MNMs. For example, during the preparation of an antibacterial motor, Delezuk et al. used three polymers, namely, PLGA, Alg, and Chi.^[^ ^68^ ^]^ A uniform nonpolar PLGA was used to protect Mg engine from its conversion to MgO. Alg provided an electrostatic interaction with the antibacterial positively charged Chi polymer (Figure 4f). Besides polymers, aptamer (RNA, ssDNA, or peptide molecules), protein (antibodies and some enzymes), and cell membranes have also been reported in functionalizing MNMs for biomedical applications.^[^ ^65^ ^]^ Therefore, the functionalization strategy should be chosen precisely according to the constituents of the motor and the intended application.^[^ ^33^ ^]^

**Figure 5:**
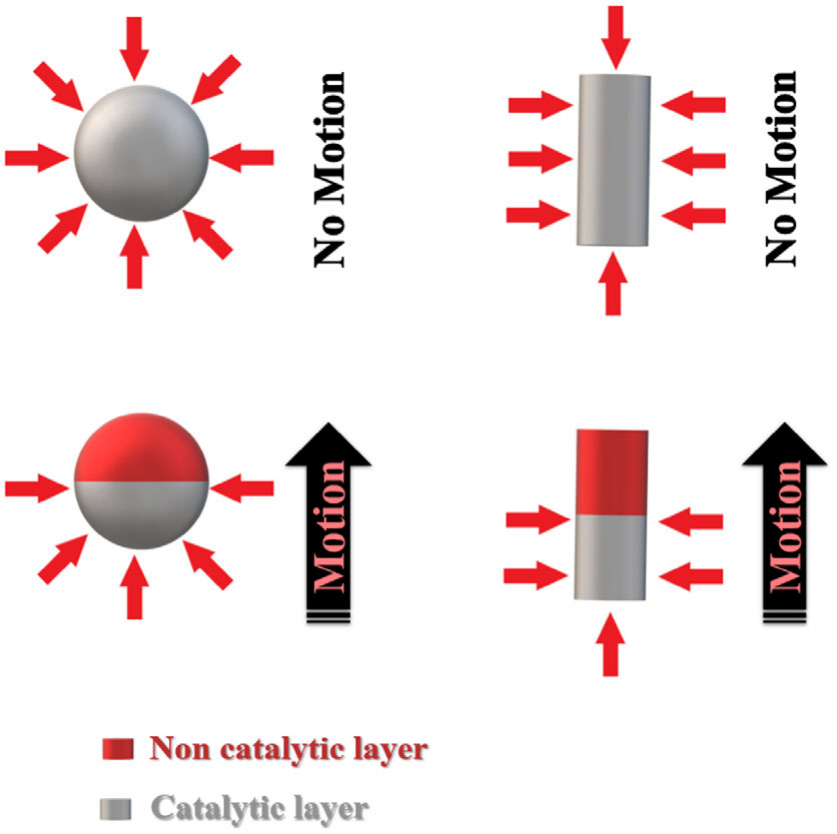
The effect of asymmetry on the propulsion mode.

It is worth noting that, due to the limitation of a low‐Reynolds‐number fluid, MNMs should provide a degree of asymmetry, either in shape or composition.^[17o]^ Therefore, structural asymmetry should be considered during the preparation and functionalization of MNMs. Such asymmetry is the main guarantee of obtaining directed propulsion.^[^ ^37^ ^]^ In an asymmetric motor, one part acts as a matrix (also known as the motor framework) and the other serves as the motor engine. If the active sites on the micro‐/nanomaterials are uniformly distributed over the surface, bubbles would be formed around the entire surface uniformly, potentially reducing the speed and increasing the orientation difficulty of the motor. As shown in **Figure** **5**, the asymmetric design leads to the formation of gas bubbles on one side of the material, and the movement direction is on the opposite side.

As a result of their unique autonomous motion behavior, tiny size, and ability to perform specific and advanced tasks, MNMs have been comprehensively used in different fields of applications, including biomedical applications (disease diagnosis,^[^ ^71^ ^]^ cargo transfer,[^17^, ^72^] drug delivery,^[^ ^14^, ^54^, ^73^ ^]^ cancer therapy,^[^ ^74^ ^]^ surgery,^[^ ^15^ ^]^ wound healing^[^ ^75^ ^]^), analytical sensing,^[^ ^31^, ^42^, ^76^ ^]^ environmental pollutant removal (organic pollutants,[^17^, ^77^] heavy metals,^[^ ^12^, ^17^, ^77^, ^78^ ^]^ bacterial contaminants,^[^ ^13^, ^68^, ^79^ ^]^ microplastics,[^17^, ^42^, ^58^, ^80^] biological warfare agents,[^17^, ^81^] and oil droplets[^58^, ^77^, ^82^]) energy generation,^[^ ^83^ ^]^ security and defense,^[^ ^84^ ^]^ and on‐the‐fly chemistry applications.^[^ ^85^ ^]^

### 2.3 Propulsion Approaches of MNMs

**Figure 6:**
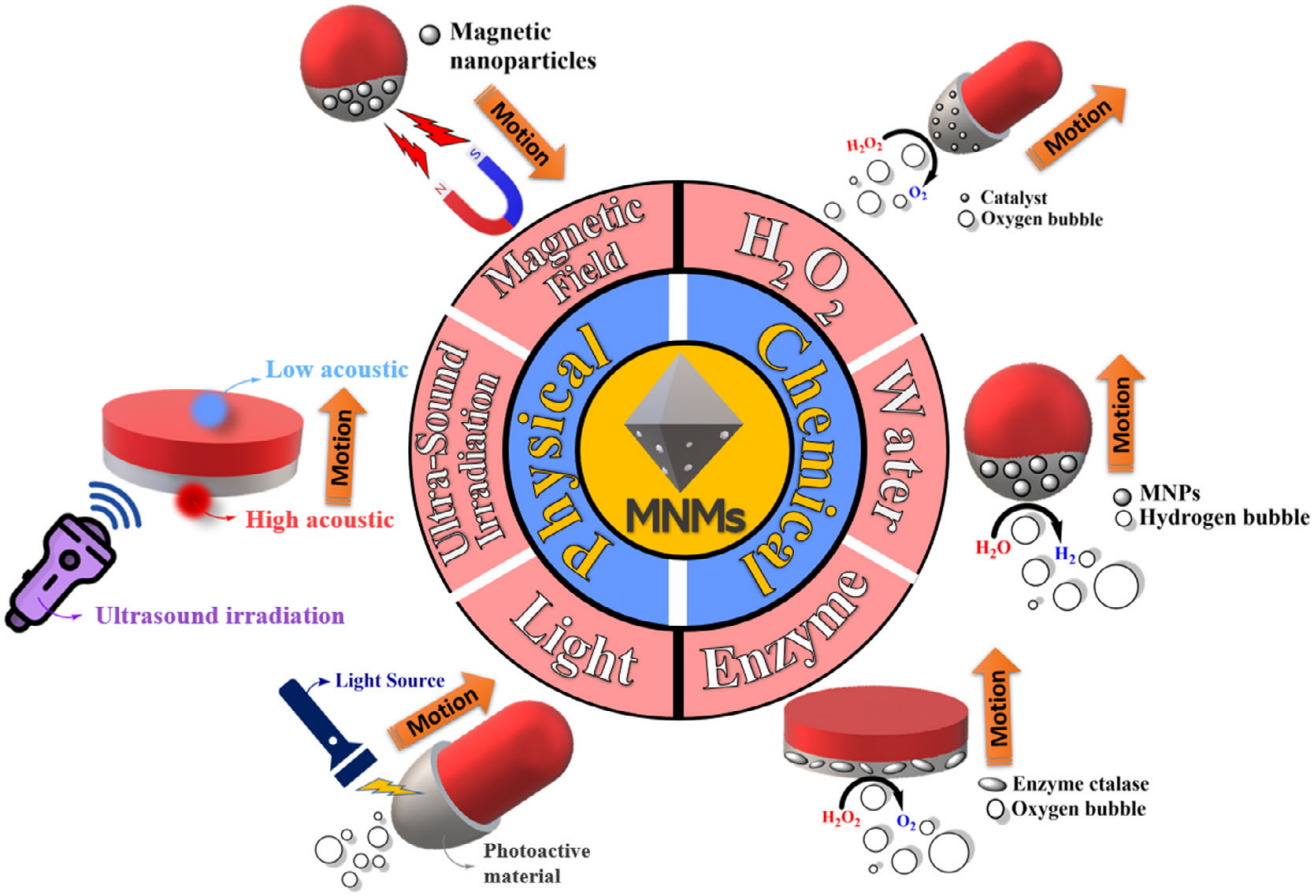
A general overview of the different energy sources of both chemical and physical propulsion of MNMs.

Machines artificially developed by human beings, like car engines, or naturally occurring systems (including the movement of adenosine triphosphate (ATP), sperm cells, and some microorganisms like the volvox algae), always have a secret behind their motion. Inspired by these machines, the movement of micro‐/nanomaterials also requires a source of energy, which can be either a chemical or an external physical form.^[^ ^76^ ^]^ **Figure** **6** summarizes some of the energy sources used by MNMs and the direction of movement.

MNMs can be classified into two types, chemically operated and physically driven MNMs. The chemically operated MNMs use chemicals in the surrounding environment and convert them into a source of motion, mainly in the form of gas bubbles.^[^ ^39^ ^]^ These chemicals, also known as the motor fuel, include hydrogen peroxide (H_2_O_2_),^[^ ^86^ ^]^ enzymes,^[^ ^87^ ^]^ sodium borohydride,[^83^, ^88^] hydrazine,^[^ ^89^ ^]^ histamine,^[^ ^90^ ^]^ halogens,^[^ ^91^ ^]^ acids and bases,^[^ ^92^ ^]^ and even water.^[^ ^93^ ^]^

**Figure 7:**
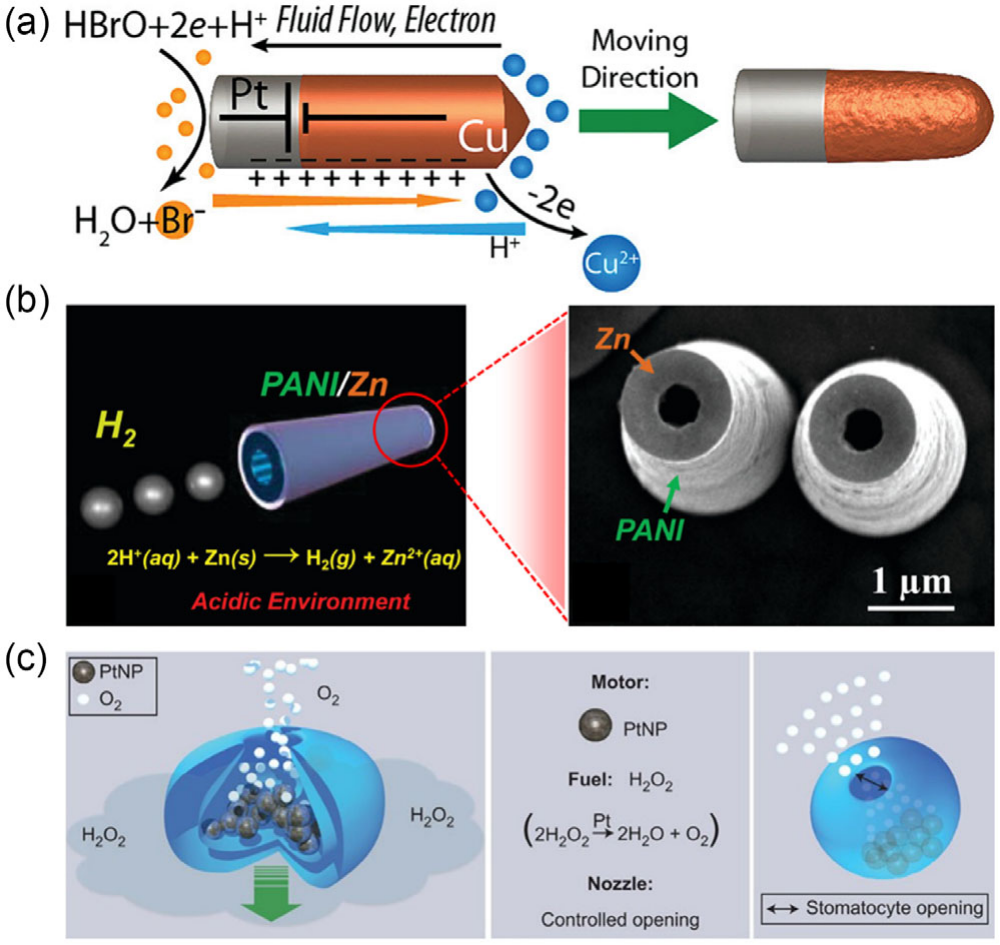
Design and mechanism of propulsion of different MNMs. a) bimetallic Cu–Pt nanorod. Adapted with permission.^[^ ^91^ ^]^ Copyright 2011, American Chemical Society. b) Tubular polyaniline (PANI)/Zn microrockets. Adapted with permission.^[92a]^ Copyright 2012, American Chemical Society. c) Pt‐entrapped inside stomatocytes polymer. Adapted with permission.^[^ ^95^ ^]^ Copyright 2012, Springer Nature.

For example, Liu and Sen reported a bubble‐free asymmetrical rod‐like nanomotor of Cu–Pt that could flow in dilute aqueous solutions of Br_2_ and I_2_.^[^ ^91^ ^]^ The rod is powered by the self‐diffusiophoresis mechanism resulting from the redox reactions occurring at the metallic surface, as illustrated in **Figure** **7**a. The surface redox reaction could be considered as a short‐circuited galvanic cell. Due to its self‐electrophoresis propulsion mechanism, nanowire motors cannot operate in a high ionic strength environment.^[^ ^94^ ^]^ To solve such a problem, Gao et al. reported a hydrogen bubble‐propelled Zn‐based tubular polyaniline microrocket that could be powered in a strongly acidic medium.^[92a]^ The motion was ascribed to the continuous thrust of hydrogen gas (H_2_) by the spontaneous oxidation–reduction reaction that took place between the Zn surface and the acid protons (Figure 7b). On the other hand, bubble‐propelled catalytic micromotors are propelled by the gas thrust generated from the catalytic decomposition of the fuel. For instance, Wilson et al. reported the autonomous motion of platinum‐loaded stomatocytes.^[^ ^95^ ^]^ The bowl‐shaped polymer stomatocytes contained stable nanocavities in which Pt NPs could be entrapped. The motor was able to propel by oxygen nanobubbles detached from the motor due to the catalytic decomposition of H_2_O_2_, as illustrated in Figure 7c.

On the contrary, the physically driven (or fuel‐free) MNMs are based on using external physical stimulus as an energy source.[^17^, ^96^] Different physical energy sources have been applied to MNMs, such as light radiation,[^17^, ^97^] ultrasound waves,^[^ ^98^ ^]^ temperature gradients,^[^ ^99^ ^]^ and electric and magnetic fields.[^28^, ^29^]

Light is one of the most exciting and considerably promising energy sources to power MNMs due to its wireless and easily controllable properties.^[17l]^ Light‐driven MNMs are based on the existence of photochromic, photothermal, photosensitive, or photocatalytic materials as one of the motor's components.^[^ ^96^ ^]^ Under the effect of light illumination, the motor can respond either by changing their molecular structure, producing chemical ions, generating thermal energy, or generating charge carriers.

**Figure 8:**
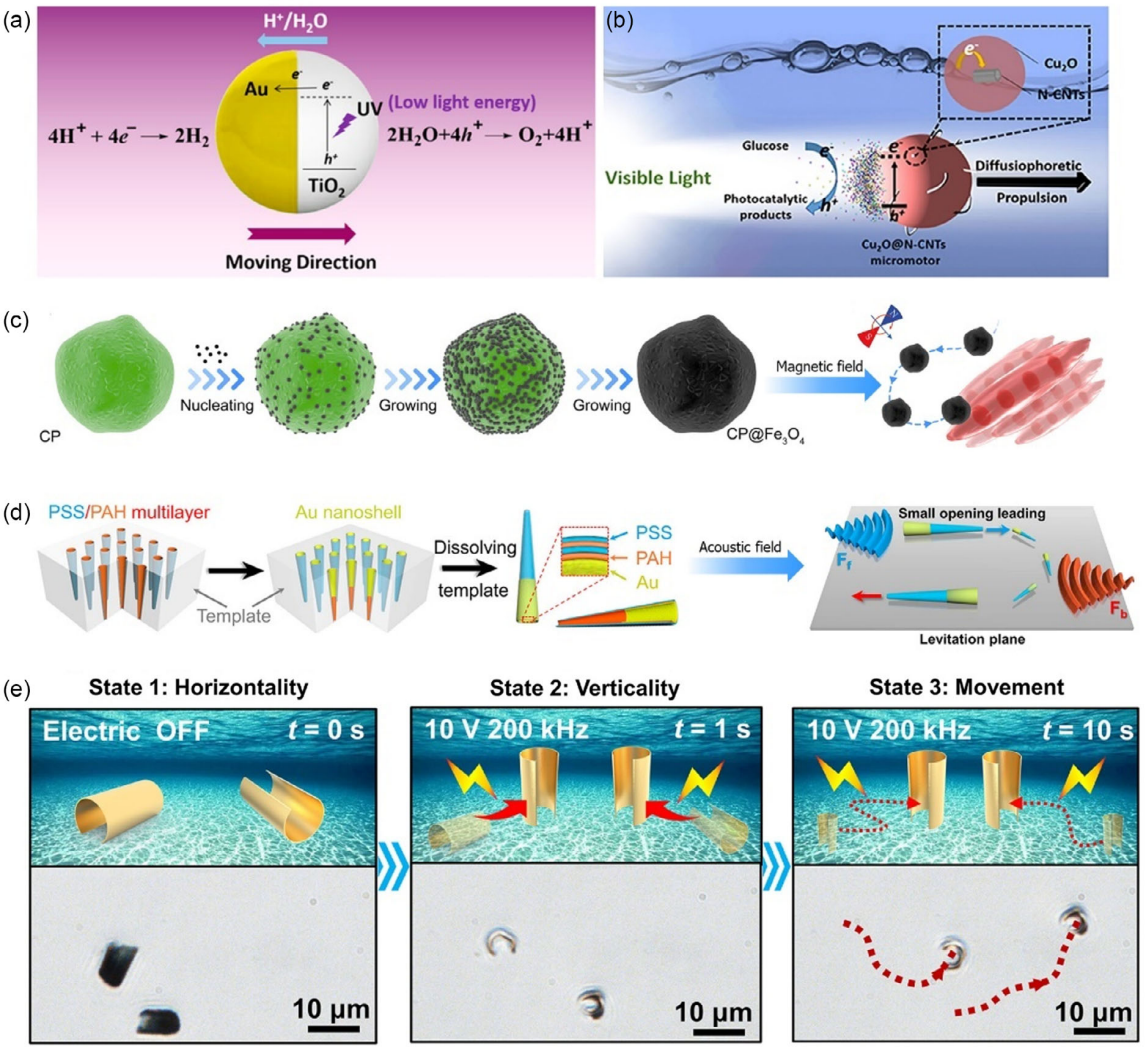
a) Structure of the catalytic TiO_2_–Au motor and the corresponding chemical reactions that occurred under UV light irradiation. Adapted with permission.^[^ ^100^ ^]^ Copyright 2015, American Chemical Society. b) Schematic of propulsion mechanism of light‐driven Cu_2_O@N‐CNT sphere micromotors. Adapted with permission.^[^ ^101^ ^]^ Copyright 2019, American Chemical Society. c) Synthesis and steering effect of CP@Fe_3_O_4_. Adapted with permission.^[^ ^102^ ^]^ Copyright 2022, American Chemical Society. d) Synthesis and controllable movement of AuNS‐functionalized PSS/PAH under the effect of acoustic field. Adapted with permission.^[^ ^105^ ^]^ Copyright 2019, American Chemical Society. e) Timelapse snapshots of the motion behavior of DGMs in the presence and absence of an electric field. Adapted with permission.^[106b]^ Copyright 2022, Elsevier.

Photocatalyst‐based MNMs are among the most widely studied light‐propelled motors due to their photochemical stability and fast response to light illumination. In this context, Dong et al. reported a Janus motor of TiO_2_–Au that can be efficiently light propelled in pure water without adding an external fuel.^[^ ^100^ ^]^ TiO_2_, as one of the most commonly studied photocatalysts, was coated with one hemisphere Au metal to provide the Janus structure. Upon light irradiation, charge separation occurred within the bands of TiO_2_ followed by electron migration from its conduction band (CB) to the Au hemisphere. Protons, generated from water oxidation, migrated to the Au, causing a fluid flow and generating a slip velocity to propel the motor forward with the TiO_2_ hemisphere (**Figure** **8**a). Cuprous oxide (Cu_2_O) is a well‐known photocatalyst with excellent photocatalytic properties under visible light irradiation. However, its high electron‐hole recombination reduces its photocatalytic activity. Combining Cu_2_O with other semiconductors is a feasible strategy to overcome this limitation. In light of this, Wang et al. built asymmetric glucose‐fuelled Cu_2_O@N‐doped carbon nanotubes (Cu_2_O@N‐CNTs) as an efficient visible light‐activated micromotor.^[^ ^101^ ^]^ The carbonaceous material (i.e., N‐CNTs) accepted the photoinduced electrons from the CB of TiO_2_, enhancing the charge separation. Under visible light illumination, photocatalytic decomposition of glucose occurred by both holes and electrons of the composite, generating different products (e.g., glyceraldehyde, formic acid, hydrogen). The product concentration gradient around the motor provided the driving force for a directional diffusiophoretic propulsion of the motor (Figure 8b).

MNMs powered by magnetic fields have gained significant research attention due to their remote activation, control, and navigation. In this class of MNMs, the motor should contain magnetic NPs, either covering its surface or being entrapped in its microcavities. Liu et al. reported magnetic biohybrid microswimmers by dip coating Fe_3_O_4_ NPs, with their superparamagnetic properties, by the *cholera* microalgae (CP).^[^ ^102^ ^]^ Under the wireless actuation of an external magnetic field, the magnetic micromotor could navigate and follow complex trajectories and approach a single targeted C2C12‐derived myotube (Figure 8c). Similarly, Yan et al. reported helical microswimmers from magnetite (Fe_3_O_4_) and *Spirulina* microalgae for imaging‐guided therapy.^[^ ^103^ ^]^ The inherent fluorescence properties of microalgae allowed in‐vivo imaging and remote diagnosis, whereas the magnetite NPs were chosen as the magnetic part of the motor.

Metallic microrods were reported to propel and rotate in water and solutions of high ionic strength under the effect of ultrasonic standing waves in the MHz frequency.^[^ ^104^ ^]^ In this context, Wang et al. introduced an ultrasound‐driven gold‐nanoshell‐functionalized polymer nanoswimmer (AuNS‐functionalized PSS/PAH).^[^ ^105^ ^]^ Upon an external acoustic field, the metallic NPs utilized a stable standing wave region in the acoustic chamber from the external acoustic field to cause the motor to be suspended in the levitation plane and showed a directional motion (Figure 8d). Moreover, electrically powered micromotors offer various advantages for the motion regime, including ease of operation, high mobility in aqueous electrolytes, and remote controllability, in addition to their biomedical applications.^[^ ^106^ ^]^ Zhuang et al. synthesized a defective golden micromotor (DGMs), which provided a locally asymmetric field gradient through the topological defects.^[106b]^ The motor was prepared by a template electrodeposition of gold over polycarbonate membrane. Under an AC electric field, the motor exhibited two types of motion behaviors (Figure 8e), namely, self‐dielectrophoresis (sDEP) and induced‐charge electrophoresis (ICEP). In the case of sDEP, the localized electric gradient formed at the two regions of the motor, with different dielectric constant, tends to interact with the induced dipole moment and propel the motor forward. In contrast, ICEP resulted in the induced‐charge electro‐osmosis flow occurring at lower frequencies.

Although the large variety of chemicals used as fuel sources make MNMs promising candidates for different applications, a fuel‐free propulsion approach is considered a safer and nontoxic way to transport these micro‐/nanomaterials in vivo. It is also worth mentioning that the nanomaterial, to be used in MNMs, should be chosen to be susceptible and directed by the energy source used.

## 3 Conception of MOF‐MNMs

**Figure 9:**
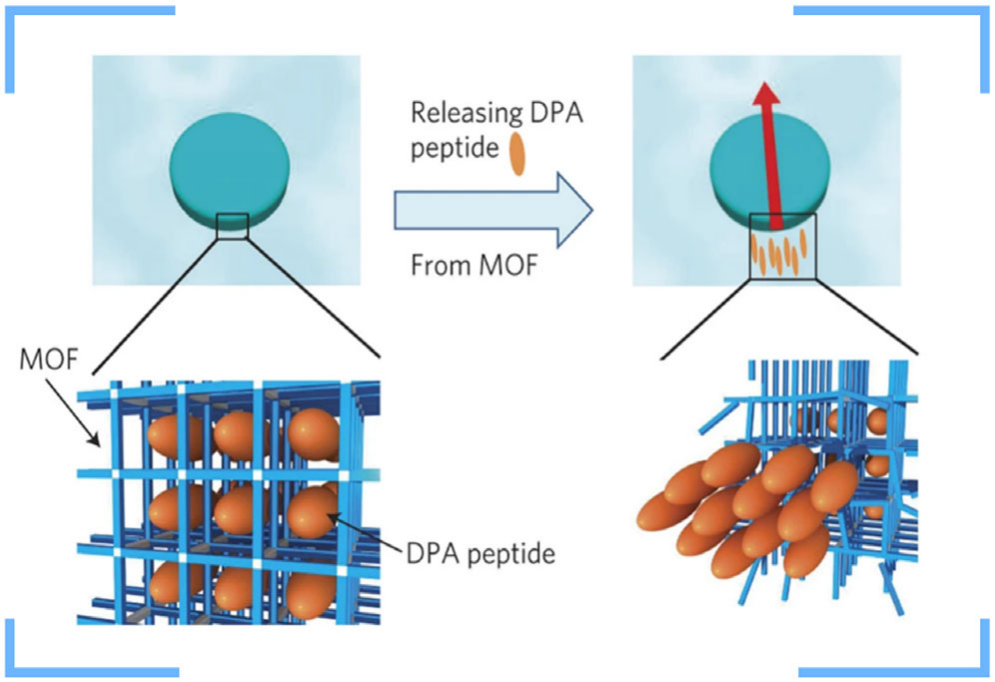
Autonomous movement of the MOF [Cu_2_(TPA)_2_(TED)]*n* resulted from the release of DPA peptides from the pores. Adapted with permission.^[^ ^107^ ^]^ Copyright 2012, Springer Nature.

In 2012, Kitagawa et al. reported the first MOF‐based MNM, based on the DPA‐incorporated biomolecular MOF [Cu_2_(TPA)_2_(TED)]_n_ (where TPA: terephthalic acid, TED: triethylenediamine, and DPA: diphenylalanine).^[^ ^107^ ^]^ The motion of this MOF‐MNM was attributed to the release of peptide molecules from the MOF pores. When injected in an ethylenediaminetetraacetic acid (EDTA) solution, the MOF is partially decomposed, releasing DPA from the pores in a highly ordered stream. The released DPA caused a tension gradient across the surface of MOF, which resulted in a strong propulsion of the MOF‐MNM via the Marangoni effect (**Figure** **9**). Later, in 2017, Grzybowski et al. reported that the motion efficiency of MOF‐MNMs depends on the chemical nature of the fuel incorporated inside the pores and the wettability of the MOF surface. In addition, the motor can be refueled several times.^[^ ^108^ ^]^

**Figure 10:**
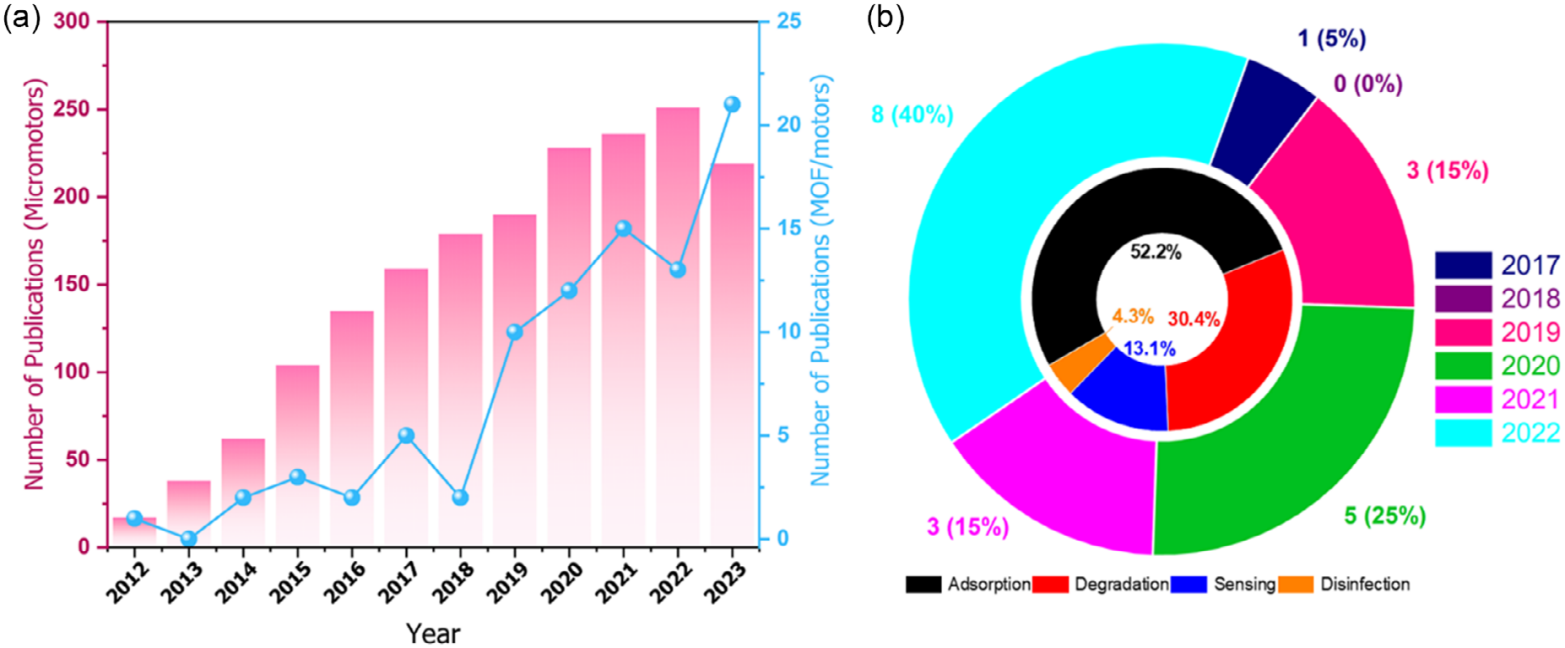
a) The number of articles about MNMs and MOF‐based motors published annually from 2012 to 2022, collected from Web of Science (keywords: “micromotors” and “MOF * motors”). b) Distribution of the published articles on MOF‐MNMs from 2017 to 2022 in each year (outer circle) and in different water‐related applications, including adsorption, degradation, sensing, and disinfection (the inner circle).

After this discovery, MOF‐MNMs have been explored in various applications, including biomedical applications,^[^ ^46^ ^]^ environmental rehabilitation,^[^ ^45^ ^]^ analytical sensing,^[^ ^109^, ^110^ ^]^ and others,^[^ ^111^ ^]^ resulting in a noticeable increase in the number of published articles about MOF‐MNMs (**Figure** **10**a).

Depending on the role of the MOF in the MNM, the MOF‐MNMs can be classified into two classes: in the first class, the MOF acts as a carrier for a rotor, motor, or machine inside its architectures,^[^ ^112^ ^]^ while in the second one, the MOF itself acts as either a motor or a part of a motor.

The articles published from 2017 to 2022 about MOF‐MNMs for wastewater treatment are distributed as in the pie chart shown in Figure 10b. The number of articles increased gradually from one article in 2017 to eight articles in 2022. In addition, the adsorption application to remove different pollutants came in the first rank and accounted for more than 50% of the applications, whereas water disinfection represented the least explored application.

**Scheme 1:**
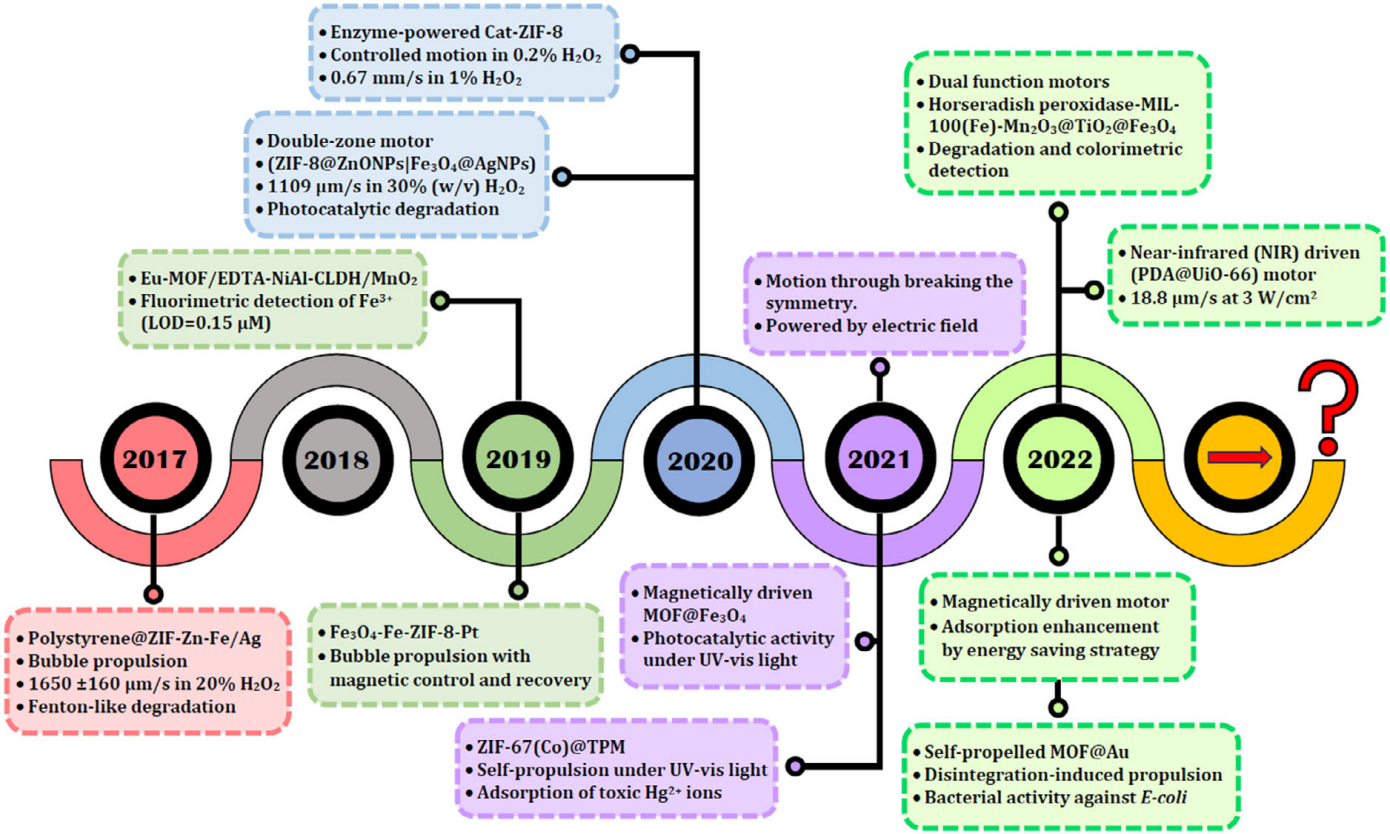
Timeline showing the critical developments in MOF‐MNMs for water treatment over 6 years, from 2017 to 2022.

The timeline of the critical development of MOF‐based MNMs for water treatment throughout this period is illustrated in **Scheme** **1**. In 2017, an ultrafast motor with a maximum speed of 1650 μm s^−1^ was reported. Later, in 2019, micromotors were utilized for sensing and removing toxic metals from a hazardous radioactive environment. In 2020, an enzymatically powered motor of Cat‐ZIF‐8 was prepared. It showed a maximum speed of 0.67 mm s^−1^, comparable to the motors decorated with noble metal NPs (MNPs), like Ag and Pt, and the motor exhibited a good speed even at a relatively low fuel concentration of 0.2% H_2_O_2_. Moreover, MOF‐MNMs powered by AC electric field through breaking the symmetry were discovered. Fuel‐free light‐driven motors were introduced and applied to remove heavy metal ions. Additionally, in 2022, colorimetric detection was introduced for determining organic compounds. This was in addition to water disinfection achieved with fuel‐free self‐propelled MOF@Au motors.

The structure of the motors, mode of propulsion, maximum recorded speed, application, as well as action mechanism of all MOF‐MNMs reported for water treatment are summarized in **Table** **1**.

**Table 1:** MOF‐MNMs reported within 2017–2022 for water remediation, containing motor structure, maximum recorded speed, propulsion power, water pollutant, method of decontamination, and removal mechanism.

Motora)			MRS	Propulsion	Analyte	Application	Mechanism	References
ZIF‐8@γ‐Fe_2_O_3_/γ‐Al_2_O_3_/MnO_2_ magnetic tubular micromotor			ZIF‐8_MEOH_‐M, 150 μm s^−1^ ZIF‐8_DMF_‐M, 105 μm s^−1^ in 5% H_2_O_2_	O_2_ propulsion by the decomposition of H_2_O_2_ over MnO_2_.	CR Doxycycline (DOC)	Adsorption CR *Q*_max_ = 394 mg g^−1^ DOC *Q*_max_ = 242 mg g^−1^	CR: electrostatic interaction with the zinc sites and the *π*–*π* stacking interaction. DOC: *π*–*π* stacking interaction.	[25]
Eu‐MOF/EDTA‐NiAl‐CLDH‐MnO_2_ hollow tubular micromotor			56.9 ± 5.5 μm s^−1^ in 5 wt% H_2_O_2_	O_2_ propulsion by the decomposition of H_2_O_2_ by MnO_2_.	Fe^3+^ ions	Adsorption (80% after 2 h, 100% after 8 h) *Q*_max_ = 112 mg g^−1^ Detection LR: 0–0.2 mm LOD: 0.15 μm	Strong interaction between Fe^3+^ and EDTA. Competition between Fe^3+^ and the motor to absorb the excitation light, in addition to the possible electron transfer from the excited Eu‐MOF to the half‐filled 3*d* orbital of Fe^3+^.	[109]
MnFe_2_O_4_@MIL‐53@UiO‐66@MnO_2_ nanomotors			N/A	O_2_ propulsion by the decomposition of H_2_O_2_ by MnO_2_.	Pb^2+^ and Cd^2+^ ions	Adsorption *Q*_max_(Pb^2+^) = 1018 mg g^−1^ *Q*_max_(Cd^2+^) = 440.8 mg g^−1^	Electrostatic interaction, partial metal exchange with the motor's metals, and chemical interaction with the carboxylic acid groups	[126]
Fe_3_O_4_–Fe‐ZIF‐8‐Pt			860 ± 230 μm s^−1^ in 5 wt% H_2_O_2_	O_2_ propulsion by the decomposition of H_2_O_2_ by Pt.	U(VI) ions	Adsorption 96% within 1 h *Q*_max_ = 384 mg g^−1^	Adsorption on the porous structure of the motor, in addition to the reduction, followed by immobilization, of U(VI) to U(IV) by the effect of Fe^2+^ in H_2_O_2_.	[120]
Cat‐ZIF‐8			≈0.67 mm s^−1^ at 1% H_2_O_2_	O_2_ propulsion by the decomposition of H_2_O_2_ by the catalase enzyme.	Heavy metal ions Ce, Cu, Co, Mn, and Ni ions Perfluorooctanoic acid (PFOA)	Adsorption Removal% Ce(99.9), Cu(88.62), Co(47.19), Mn(32.9), Ni(33.56), and PFOA (84.53).	The metals were adsorbed on the acid‐basic sites of ZIF‐8, which is enhanced by the circular propulsion mode. PFOA was adsorbed on the pores of ZIF‐8, accompanied by strong adsorption energy.	[117]
ZIF‐67(Co)@3‐trimethoxysilylpropyl methacrylate (TPM) colloidal motor (MOF‐TPM)			UV light only 22.5 ± 2 μm s^−1^ UV + 1%H_2_O_2_ ≈43 μm s^−1^	Light‐powered and fuel‐free propulsion (UV and Visible)	Hg^2+^, Pb^2+^, Cd^2+^, and Cu^2+^ ions	Adsorption Hg^2+^ (≈90% within 40 min) Pb^2+^, Cd^2+^, and Cu^2+^ (55–75% within 40 min)	Physical adsorption on the MOF surface and chemical through an electrostatic interaction with the nitrogen atom of the 2‐methylimidazole linker	[26]
UiO‐66 MOF deposited on mesoporous polydopamine. (PDA@UiO‐66)			18.8 μm s^−1^ at 3 W cm^−2^ power density	Near‐infrared (NIR) light‐based propulsion	MB dye	Adsorption (78% within 40 min) *Q*_max_ = 134 mg g^−1^	The adsorption is due to *π*–*π* stacking interaction between benzene rings of both MB and terephthalic acid of the UiO‐66.	[27]
A series of polyhedral MOF micromotors		MIL‐96 (M_1_) UiO‐66 (M_2_) MIL‐88B (M_3_) and ZIF‐8 (M_4_)	11.4 μm s^−1^ 4.27 ± 0.43 μm s^−1^ 2.94 ± 0.24 μm s^−1^ Null	Propulsion by (AC) electric field	Fluoride (F^−^) ions	Adsorption (1 mg of the motor adsorbed 0.004 mg of fluoride in 0.5 h)	The fluoride ions are adsorbed in the cavities of the MOF motors.	[29]
Rod‐like Fe_3_O_4_@Ce‐MOF nanomotor			Forward speed 16.69 ± 1.10 μm s^−1^ rotation speed 498 ± 113.6 rpm at 10 Hz	Magnetically driven motor	MB Oxytetracycline (OTC)	Adsorption MB (100% after 10 min) *Q*_max_ in tumbling mode is 13.14 mg g^−1^, and in rotation mode is 10.56 mg g^−1^. OTC (24.6%)	Molecular entrapping inside the porous structure of the motor.	[28a]
Enzyme catalase@ UiO‐type Zr‐MOF			In 1.5% H_2_O_2_, the speed was 3.56 ± 0.56 body‐lengths s^−1^ and lasted for 7.0 ± 0.4 min	O_2_ bubble propulsion through the enzymatic decomposition of H_2_O_2_.	Rhodamine B (RhB)	Adsorption (51.0 ± 2.7% of RhB after 5 min of incubation)	Adsorption of dye molecules on the remaining unoccupied micro‐ and mesopores of the MOF.	[118]
ZIF‐67 micromotor with spherical (S) and dodecahedral (D) morphologies			In 10%H_2_O_2_, ‐ZIF‐67‐D is 61.39 μm s^−1^ and ZIF‐67‐S is 55.82 μm s^−1^ The motion lasted for 90 min	O_2_ propulsion by the decomposition of H_2_O_2_ by ZIF‐67.	Methyl blue	Adsorption (The motor adsorbs almost 3 × 10^−3^ mol L^−1^ within 5 min)	The adsorption mechanism was attributed to the high surface area and porosity of ZIF‐67.	[116]
Fe_3_O_4_@NH_2_‐UiO‐66 colloidosomes decorated with Pt NPs micromotor (Fe‐UiOSomes‐Pt)			Micromotor‐C 450 ± 180 μm s^−1^ Micromotor‐P 303 ± 93 μm s^−1^ in 5 wt% H_2_O_2_	O_2_ propulsion by the decomposition of H_2_O_2_ by Pt.	Methyl orange (MO) Potassium dichromate (Cr^6+^)	Adsorption Removal efficiency after 2 h: MO (94%) Cr^6+^ (91% within 2 h)	The removal was attributed to both the electrostatic interaction, between pollutants and the motor, and the motion‐induced convection between them.	[125]
Polystyrene@ZIF–Zn–Fe/Ag (Ag–ZIF)			In 6% H_2_O_2_ 623 ± 61 μm s^−1^ In 20% H_2_O_2_ 1650 ± 160 μm s^−1^	O_2_ propulsion by the decomposition of H_2_O_2_ by Ag	Rhodamine B (RhB)	Degradation (93.1%, 150 min)	Adsorption on the surface of the motor and heterogenous Fenton oxidation.	[119]
ZIF‐8@ZnONPs|Fe_3_O_4_@AgNPs			In 30% H_2_O_2_, 1109 μm s^−1^	O_2_ propulsion by the decomposition of H_2_O_2_ by Fe_3_O_4_@AgNPs	Rhodamine B (RhB)	Degradation (98.6%, 60 min)	Adsorption on the surface of the motor Photocatalytic performance of ZIF‐8@ZnONPs Advanced oxidation caused by H_2_O_2_/UV	[130]
Fe_3_O_4_/ZIF‐67(Co) micromotor			115 μm s^−1^ at 10% H_2_O_2_	O_2_ propulsion by the decomposition of H_2_O_2_ over the motor	MB	Degradation (90%, within 1 h)	Adsorption on the surface of the motor and the synergistic Fenton effect caused by Co^2+^ & Fe^2+^ ions of the motor.	[121]
laccase@Fe‐BTC/NiFe_2_O_4_@Mn_2_O_3_ tubular micromotor			300 ± 27.5 μm s^−1^ in 5% H_2_O_2_	O_2_ propulsion by the decomposition of H_2_O_2_ over Mn_2_O_3_	MB	Degradation (100%, within 20 min)	The synergistic catalytic effect of Fenton, natural laccase enzyme, and the nanozymes (Fe‐BTC/NiFe_2_O_4_).	[131]
A series of biotemplated MOF‐based small‐scale robots MOF@Fe_3_O_4_		MIL‐100(Fe) MIL‐125NH_2_(Ti) UiO‐66(Zr) ZIF‐8(Zn)	28 μm s^−1^ at 17 Hz magnetic field frequency	Magnetically driven MOF‐based robots (MOFBOTs)	Rhodamine B (RhB) Cancer therapeutic drug Doxorubicin (DOX)	Degradation (98%, within 60 min by using MIL 125NH_2_ @MOFBOTs) Drug delivery	Adsorption of dye molecules in the MOF pores and photo‐catalytic degradation by the hydroxy radicals formed during the photocatalysis.	[28b]
Fe_3_O_4_@MnO_2_@HKUST‐1			99 ± 2 μm s^−1^ in 5% H_2_O_2_	O_2_ propulsion by the decomposition of H_2_O_2_ over MnO_2_ It can be magnetically driven	Hydroquinone (HQ)	Degradation (96.92% after 60 min) Detection LR: 1–280 μm LOD: 0.94 μm	Adsorption of HQ on the HKUST‐1 MOF, followed by degradation of the adsorbed HQ by the (.OH) generated from the reaction between H_2_O_2_ and the motor. The motor oxidizes TMB and forms a deep blue color decolorized upon reaction with HQ.	[132]
Horseradish peroxidase‐MIL‐100(Fe)–Mn_2_O_3_@ TiO_2_@Fe_3_O_4_ (HRP‐MIL@TiO_2_‐MMT)			786 ± 37.5 μm s^−1^ in 7% H_2_O_2_	O_2_ propulsion by the decomposition of H_2_O_2_ over manganese sesquioxide (Mn_2_O_3_)	Hydroquinone (HQ)	Degradation (Under simulated sunlight: 91%, within 70 min) Detection LR: 2–240 μm LOD: 1.84 μm	Photo‐Fenton degradation Oxidation of TMB by the micromotor to form ox‐TMB with a blue color. Decolorization of the blue color of ox‐TMB in the presence of HQ.	[110]
A series of MOF@Au	ZIF‐90 ZIF‐67 ZIF‐8 ZIF‐5 UiO‐66		15.9 μm s^−1^ 17.2 μm s^−1^ 9.7 μm s^−1^ 5.6 μm s^−1^ Brownian motion	Self‐propulsion capability from the MOF‐disintegration in water	*Escherichia coli* (*E. coli*)	Disinfection (96.3% death rate by ZIF‐90@Au and 97.4% by ZIF‐67@Au micromotors)	The metal ions killed *E. coli* by inhibiting the action of some enzymes, leading to bacterial death.	[30]

a) MRS: Maximum recorded speed, *Q*_max_: maximum adsorption capacity, LR: linear range, LOD: limit of detection, N/A: not available.

## 4 Fabrication of MOF‐MNMs

The fabrication of MOF‐MNMs is highly dependent on the role of MOF in the MNMs. In general, we have two types of MOF‐MNMs: self‐engine MOF‐MNMs and MOF‐decorated motors.

### 4.1 Self‐Engine MOF‐MNMs

In this class of MOF‐MNMs, the MOF simultaneously acts as the framework and the motor engine; therefore, the MOF should be able to propel in the reaction environment. In this context, it is important to consider the different synthetic approaches of MOFs, like solvothermal/hydrothermal,^[^ ^113^ ^]^ microwave‐assisted,^[^ ^114^ ^]^ and electrochemical synthesis.^[^ ^115^ ^]^

**Figure 11:**
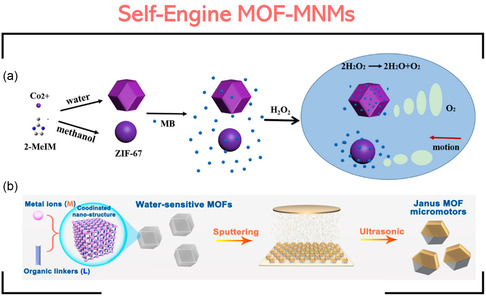
Synthesis of self‐engine MOF‐MNMs. a) Synthesis and propulsion of ZIF‐67 motors. Adapted with permission.^[^ ^116^ ^]^ Copyright 2020, Springer Nature. b) Synthesis of MOF@Au motors. Adapted with permission.^[^ ^30^ ^]^ Copyright 2022, American Chemical Society.

For example, Chen et al. fabricated a ZIF‐67 micromotor using a direct, one‐step method from the reaction of CoSO_4_·7H_2_O metal source with 2‐methylimidazole (2‐MeMI) ligand in water (or methanol) at room temperature. The ZIF‐67 micromotor can catalyze H_2_O_2_ decomposition producing O_2_ gas, as illustrated in **Figure** **11**a.^[^ ^116^ ^]^ In this example, the MOF did not require any modification or functionalization to work as MNM. However, in some cases, surface coating might be required to break the symmetry and provide some asymmetry. For instance, a series of self‐fuelled motors, MOF@Au (where MOF: ZIF‐90, ZIF‐67, ZIF‐8, MOF‐5, and UiO‐66), were fabricated and applied against *Escherichia coli (E. coli)* bacteria.^[^ ^30^ ^]^ The MOFs were first prepared by mixing the metal and ligand, either at room temperature or in the autoclave. The second step involved coating the prepared MOFs with a passive Au layer to form Janus structures. Ethanolic suspension solutions of the MOFs were dropped onto clean glass slides followed by depositing the Au particles using an electron beam evaporation system (Figure 11b).

### 4.2 MOF‐Decorated Motors

For the MOF‐decorated motors, the MOF can work either as an engine carrier or as a part of the hierarchical motor structure.

#### 4.2.1 MOF as an Engine Carrier

In this context, the MOF is combined with the catalyst (referred to as an “engine”), which is generally in the form of either an enzyme (biotic or biocatalytic engine) or an artificial nanoscale material (abiotic or chemical engine).

##### Biotic MOF‐MNMs

The MOF is modified with enzyme molecules, which catalyze the fuel (e.g., H_2_O_2_) decomposition to produce the gas (e.g., O_2_) required for motor propulsion. The enzyme can be introduced into the MOF either through in situ or sequential encapsulation strategies.

**Figure 12:**
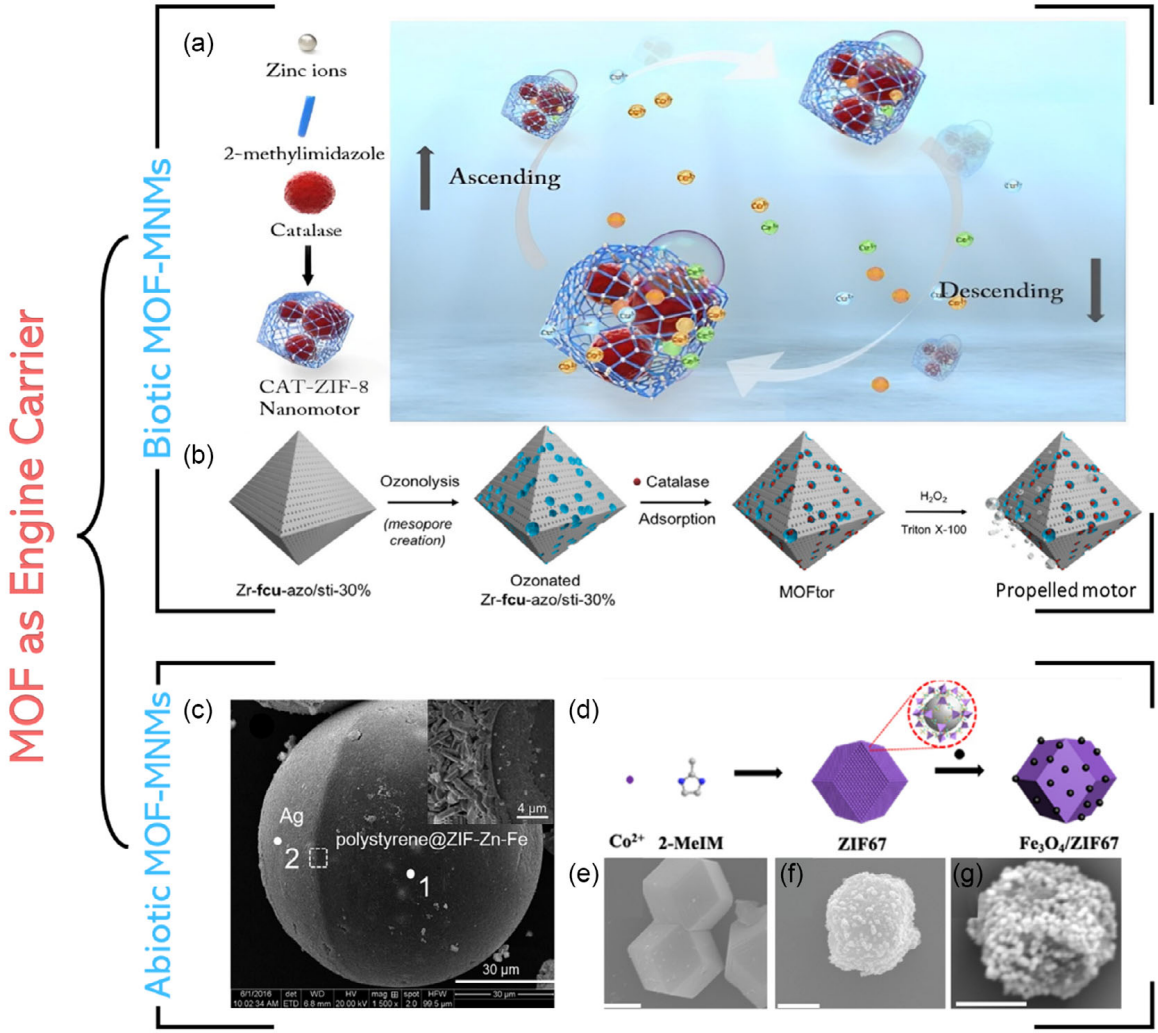
Synthesis of biotic and abiotic MOF‐MNMs. a) Synthesis and motion scheme of Cat‐ZIF‐8. Adapted with permission.^[^ ^117^ ^]^ Copyright 2020, The Royal Society of Chemistry. b) Fabrication and propulsion of catalase enzyme encapsulated Zr‐fcu‐azo/sti‐30%. Adapted with permission.^[^ ^118^ ^]^ Copyright 2020, American Chemical Society. c) SEM image of Ag‐ZIF motor. Reproduced with permission.^[^ ^119^ ^]^ Copyright 2020, Royal Society of Chemistry. d–g) Synthesis of Fe_3_O_4_/ZIF‐67(Co) and the corresponding SEM images (e: ZIF‐67& f,g: Fe_3_O_4_/ZIF‐67 micromotors). Adapted with permission.^[^ ^121^ ^]^ Copyright 2022, John Wiley and Sons.

The in situ encapsulation is based on directly incorporating the enzyme molecules during the MOF preparation. Liang et al. dissolved enzyme catalase molecules in the ligand solution (2‐MeMI) with a metal source (zinc nitrate) to give a biocatalytic MOF‐MNMs, named Cat‐ZIF‐8 (**Figure** **12**a).^[^ ^117^ ^]^ The other strategy, sequential encapsulation, involves depositing or encapsulating the enzyme molecules into a presynthesized MOF. In 2020, Sanchez et al. fabricated an enzyme‐powered micromotor from a UiO‐type Zr‐MOF.^[^ ^118^ ^]^ First, the Zr‐MOF was prepared and was then incubated in an aqueous solution of enzyme catalase for different periods to obtain enzyme‐encapsulated MOF‐MNMs, as illustrated in Figure 12b.

##### Abiotic MOF‐MNMs

The abiotic MOF‐MNMs are a class of motors in which the engine is an artificial nanoscale material, typically as MNPs (Ag^[^ ^119^ ^]^ and Pt^[^ ^120^ ^]^) or metal oxides (such as Fe_3_O_4_^[^ ^121^ ^]^). There are two main approaches to fabricate this class of MOF‐MNMs, called “ship‐in‐a‐bottle” and “bottle‐around‐Ship”.^[^ ^122^ ^]^ The former one involves building or preparing the engine into the presynthesized MOF,^[^ ^119^, ^121^ ^]^ whereas the latter strategy is based on assembling the MOF around an already prepared engine.^[28a]^

For the deposition of MNP engines into the MOF, various techniques are usually used, including chemical vapor deposition, physical vapor deposition, chemical reduction, and electrochemical deposition, as described elsewhere.^[^ ^123^ ^]^ For the “ship‐in‐a‐bottle” strategy, a spherical motor with the formula polystyrene@ZIF‐Zn‐Fe/Ag, denoted as Ag‐ZIF, was synthesized using polystyrene spherical particles.^[^ ^119^ ^]^ First, the bimetallic MOF (BMOF) ZIF‐Zn‐Fe was prepared in the presence of polystyrene particles. Then, the as‐synthesized microsphere was then coated with a catalytic hemisphere layer of AgNPs through a sputtering technique (Figure 12c).

However, Wang and co‐workers fabricated a micromotor of Fe_3_O_4_/ZIF‐67(Co) through the “bottle‐around‐ship” approach.^[^ ^121^ ^]^ The general procedure and the corresponding scanning electron microscope (SEM) images of Fe_3_O_4_/ZIF‐67(Co) are illustrated in Figure 12d–g. A solvothermally prepared Fe_3_O_4_ was dispersed ultrasonically in an aqueous solution of poly (sodium 4‐styrene sulfonate) (PSS) to give PSS‐functionalized Fe_3_O_4_ NPs. Then, ZIF‐67 was synthesized and mixed with the as‐prepared PSS‐functionalized Fe_3_O_4_ to give the micromotor Fe_3_O_4_/ZIF‐67(Co). In addition, Cai and co‐workers prepared the magnetic micromotor Fe_3_O_4_@Ce‐MOF following the same strategy but in a one‐step process.^[28a]^

#### 4.2.2 MOF‐Based Hierarchical Motors

Besides working as a self‐engine and as an engine carrier, MOF can be a part of a hierarchical motor. A hierarchical motor is composed of multiple components or subsystems that work together to achieve various tasks. In other words, each component in these motors is chosen precisely to perform a specific function.

**Figure 13:**
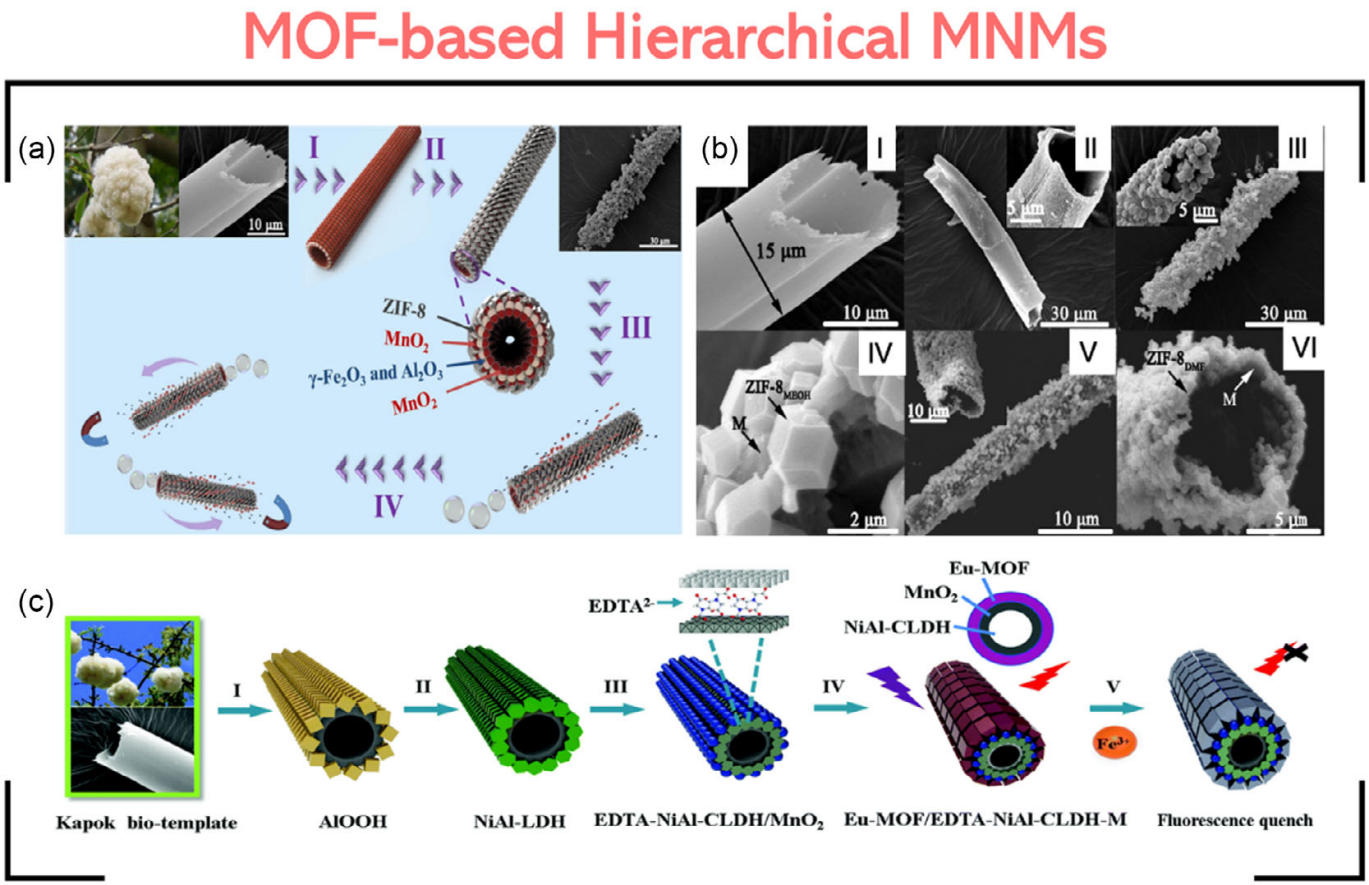
Synthesis of MOF‐based hierarchical MNMs. a,b) Synthesis of ZIF‐8@γ‐Fe_2_O_3_/γ‐Al_2_O_3_/MnO_2_ tubular motor and the corresponding SEM images (I: kapok template, II: γ‐Fe_2_O_3_/γ‐Al_2_O_3_/MnO_2_ (M), III‐IV: ZIF‐8_MEOH_‐M, and V‐VI: ZIF‐8_DMF_‐M under different magnifications). Adapted with permission.^[^ ^25^ ^]^ Copyright 2019, Elsevier. c) Synthesis of Eu‐MOF/EDTA‐NiAl‐CLDH/MnO_2_ motor and the fluorescence quenching by Fe^3+^ ions. Adapted with permission.^[^ ^109^ ^]^ Copyright 2019, The Royal Society of Chemistry.

This class of MOF‐MNMs mainly requires building the motor on a template. Li et al. built a bioinspired ZIF‐8@γ‐Fe_2_O_3_/γ‐Al_2_O_3_/MnO_2_ motor.^[^ ^25^ ^]^ First, a kapok fiber was immersed in a mixture of metal ions (Al and Fe) followed by calcination to give a hollow magnetic microtube. The magnetic tube was then dispersed in a manganese solution before being calcined again. The calcination allowed the engine, MnO_2_, to be deposited on both sides of the magnetic microtube, giving the magnetic micromotor γ‐Fe_2_O_3_/γ‐Al_2_O_3_/MnO_2_. The final step involved the immobilization of ZIF‐8 on the outer layer of the tube to give the final magnetic micromotor ZIF‐8@γ‐Fe_2_O_3_/γ‐Al_2_O_3_/MnO_2_. **Figure** **13**a,b shows the fabrication process of the motor and the corresponding SEM images, respectively.

Another template‐based motor was the fluorescent Eu‐MOF/EDTA‐NiAl‐CLDH‐MnO_2_ motor, which was assembled on the kapok fiber (Figure 13c).^[^ ^109^ ^]^ The motor comprises two layers deposited on the natural kapok fiber with a hollow tubular structure. The tube's outer layer was decorated with the fluorescent Eu‐MOF. At the same time, the inner part was coated with EDTA‐NiAl‐CLDH/MnO_2_.

## 5 Applications of MOF‐MNMs for Wastewater Remediation

This section will discuss the different MOF‐MNMs used for adsorption, catalytic degradation, and sensing, in addition to disinfection, of different water pollutants.

### 5.1 MOF‐MNMs for Adsorption

One of the most extensive applications of MOF‐MNMs involves the removal of water pollutants (such as metals, dyes, and other contaminants) through adsorption due to their outstanding surface area, high porosity, and water stability.

#### 5.1.1 Adsorption of Metal Ions

**Figure 14:**
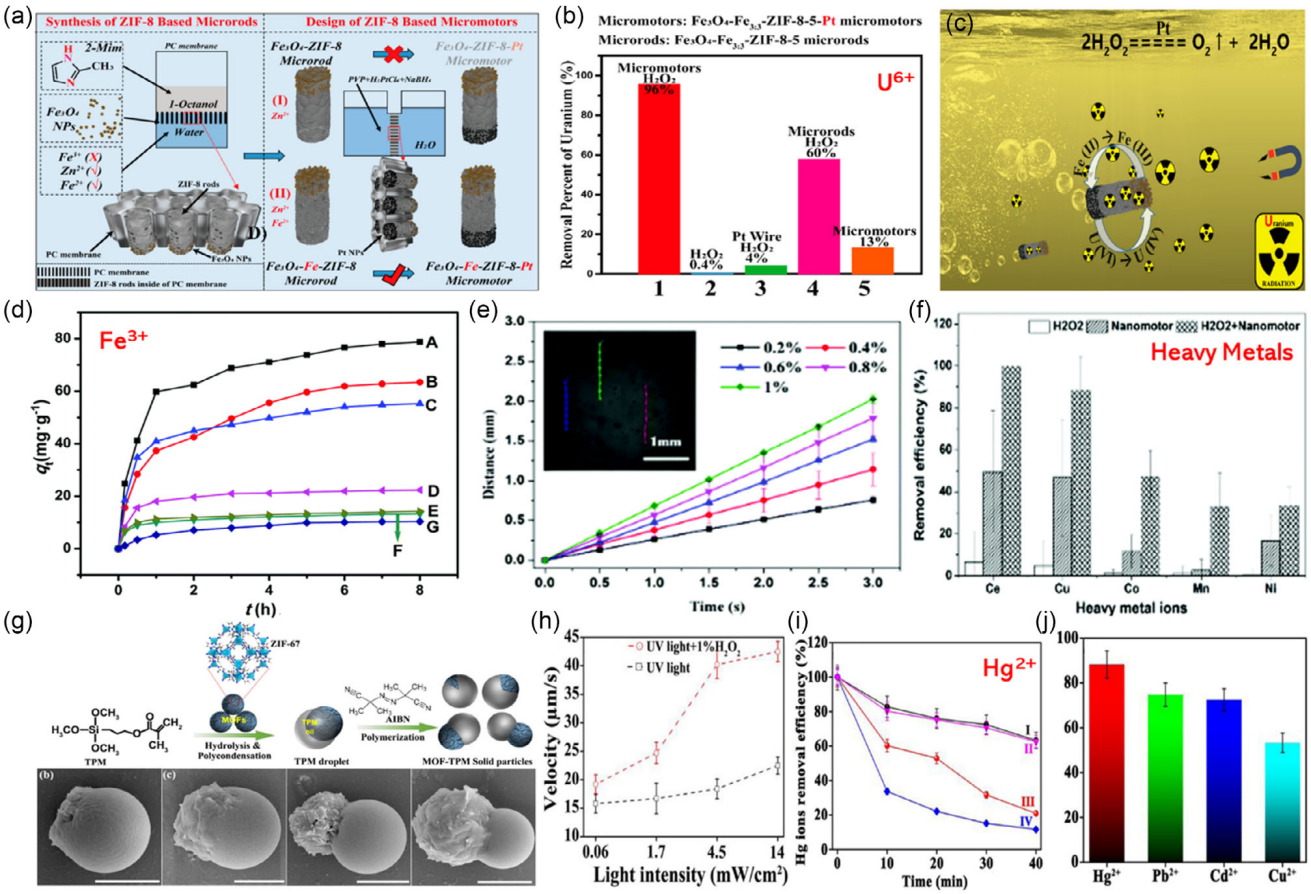
a) A schematic diagram for the synthesis of both Fe_3_O_4_‐Fe‐ZIF‐8 microrod and Fe_3_O_4_‐Fe‐ZIF‐8‐Pt micromotors, b) adsorption percentage of uranium under different conditions, and c) the proposed mechanism of the uranium removal with the Fe_3_O_4_‐Fe‐ZIF‐8‐Pt micromotor. Adapted with permission.^[^ ^120^ ^]^ Copyright 2019, American Chemical Society. d) Adsorption of Fe^3+^ by Eu‐MOF/EDTA‐NiAl‐CLDH–M (A: H_2_O_2_/stirring; B: H_2_O_2_/no stirring; C: no H_2_O_2_/stirring; D: H_2_O_2_/stirring; E: no H_2_O_2_/stirring; F: no H_2_O_2_/no stirring; G: pure Eu‐MOF/no H_2_O_2_/stirring). Adapted with permission.^[^ ^109^ ^]^ Copyright 2019, The Royal Society of Chemistry. e) Effect of [H_2_O_2_] on the motion behavior of the Cat‐ZIF‐8 motor and f) adsorption of heavy metals by Cat‐ZIF‐8 motor. Adapted with permission.^[^ ^117^ ^]^ Copyright 2020, The Royal Society of Chemistry. g) Schematic representation of the formation of a MOF‐TPM motor, h) effect of UV light intensity on the propulsion speed of the motor with/and without H_2_O_2_, i) adsorption behavior of Hg ions (I‐static sample, II‐sample/H_2_O_2_, III‐sample/UV, and IV‐sample/UV/H_2_O_2_), and j) adsorption behavior of the motor MOF‐TPM toward Hg^2+^, Pb^2+^, Cd^2+^, and Cu^2+^. Adapted with permission.^[^ ^26^ ^]^ Copyright 2021, American Chemical Society.

Owing to its self‐ and easily controllable propulsion, MOF‐MNMs were used to remove radioactive water pollutants, one of the most challenging pollutants to be handled. Pumera and co‐workers engineered a self‐propelled microtubular motor based on a BMOF Fe_3_O_4_‐Fe‐ZIF‐8‐Pt.^[^ ^120^ ^]^ The monometallic ZIF‐8(Zn) was doped by Fe^2+^ ions during the manufacturing process of both Fe_3_O_4_‐Fe‐ZIF‐8 microrod and Fe_3_O_4_‐Fe‐ZIF‐8‐Pt micromotor (**Figure** **14**a). The role of iron doping here is to improve the stability of ZIF‐8 in both H_2_O_2_ and acidic media. The motor moves due to the bubble produced from the catalytic decomposition of H_2_O_2_ by Pt. The maximum speed of the motor was 860 ± 230 μm s^−1^ in the solution of 5 wt% H_2_O_2_ and 1 wt% sodium dodecyl sulfate (SDS) surfactant. The role of the surfactant in the bubble‐propulsion motors is to decrease the surface tension at the motor surface, facilitating the bubble formation and release from the motor. Another merit of this motor is the presence of Fe_3_O_4_, which allows fast collection of the motor with the aid of a magnet and motion control through an external magnetic field. The adsorptive removal of uranium species from water has been studied using Fe_3_O_4_‐Fe‐ZIF‐8 microrod and Fe_3_O_4_‐Fe‐ZIF‐8‐Pt micromotor. Figure 14b shows that the removal percentage of uranium by the micromotor in the presence of 1% H_2_O_2_ and surfactant (case 1) is almost 96% with a maximum adsorption capacity of 384 mg g^−1^. In contrast, only 60% removal was achieved in the case of the microrods/H_2_O_2_ system without a Pt catalyst (case 4). These data proved the crucial rule of Pt NPs, as a catalyst for H_2_O_2_ decomposition, in the adsorption process. Control experiments were carried out to examine the effect of H_2_O_2_ and Pt on the adsorption process. As shown in Figure 14c, the removal mechanism of U(VI) was explained by adsorption on the porous structure of the motor, and a reduction of U(IV) by the effect of Fe^2+^/H_2_O_2_ system, followed by its immobilization on the surface of the motor.

In 2019, Li and co‐workers reported Eu‐MOF/EDTA‐NiAl‐CLDH‐MnO_2_ (abbreviated, MOF‐M) for detecting and removing Fe^3+^ ions from water.^[^ ^109^ ^]^ The motor consisted of two layers with the fluorescent Eu‐MOF (responsible for sensing Fe^3+^ ions, discussed in Section 5.3) as the outer layer whereas the inner part was coated with EDTA‐NiAl‐CLDH/MnO_2_ for iron adsorption. The motor showed an autonomous circle‐like propulsion caused by the asymmetric catalytic decomposition of H_2_O_2_ over the catalyst MnO_2_. The speed in 1 wt% H_2_O_2_ was 24.1 ± 2.8 μm s^−1^, and the maximum velocity was 56.9 ± 5.5 μm s^−1^ in 5 wt% H_2_O_2_. They studied the adsorption of Fe^3+^ by MOF‐M (Figure 14d). The maximum adsorption capacity of the motor was calculated to be 112 mg g^−1^. It was also proved that bubble propulsion produced much higher adsorption of Fe^3+^ than external stirring. The adsorption mechanism was attributed to the strong interaction between the Fe^3+^ ions and the unoccupied adsorption sites of EDTA in the motor MOF‐M.

Liang and co‐workers reported an enzyme‐powered Cat‐ZIF‐8 nanomotor.^[^ ^117^ ^]^ Because of the high selectivity and catalytic activity of the enzyme catalase, the Cat‐ZIF‐8 motor showed a highly controlled motion even at a low concentration of H_2_O_2_ of 0.2–1% (Figure 14e), and the maximum speed was almost 0.67 mm s^−1^ at 1% H_2_O_2_ concentration. The motor was applied to remove different types of water pollutants including heavy metal ions (Ce, Cu, Co, Mn, and Ni) and organic compounds (perfluorooctanoic acid, PFOA). The adsorption efficiencies (Figure 14f) of Cat‐ZIF‐8 toward Ce, Cu, Co, Mn, and Ni in Milli‐Q water within 20 min were found to be 99.9, 88.62, 47.19, 32.9, and 33.56%, respectively. The high removal efficiencies were attributed to the strong interaction of the metals on the acid–basic sites of ZIF‐8, which is enhanced by the circular motion of the motor.

Gao et al. synthesized a fuel‐free, light‐powered ZIF‐67(Co)@3‐trimethoxysilylpropylmethacrylate colloidal motor (MOF‐TPM), as illustrated in Figure 14g.^[^ ^26^ ^]^ The motor displayed a self‐propulsion under UV light, due to the light‐triggered ionic self‐diffusiophoresis. The motor speed was highly dependent on the light intensity, and the maximum velocity under a UV light intensity of 14 mW cm^−2^ was 22.5 ± 2 μm s^−1^. It was also found that the speed was almost doubled in 1% [H_2_O_2_] as an external “fuel,” reaching ≈43 μm s^−1^ (Figure 14h). Under visible light irradiation, oxidation of water molecules occurred leading to a self‐propulsion with a speed of 13.6 ± 2 and 10.4 ± 2 μm s^−1^ using both blue and green light, respectively. The motor showed a high removal of Hg^2+^ ions from water with an efficiency of ≈90% after 40 min (Figure 14i) and other metals’ (Pb^2+^, Cd^2+^, and Cu^2+^) removal efficiencies ranging from 55% to 75% (Figure 14j). The high removal efficiency was attributed to two types of adsorptions, physical on the MOF surface and chemical through the electrostatic interaction of Hg^2+^ with the nitrogen atom of the 2‐methylimidazole linker.

**Figure 15:**
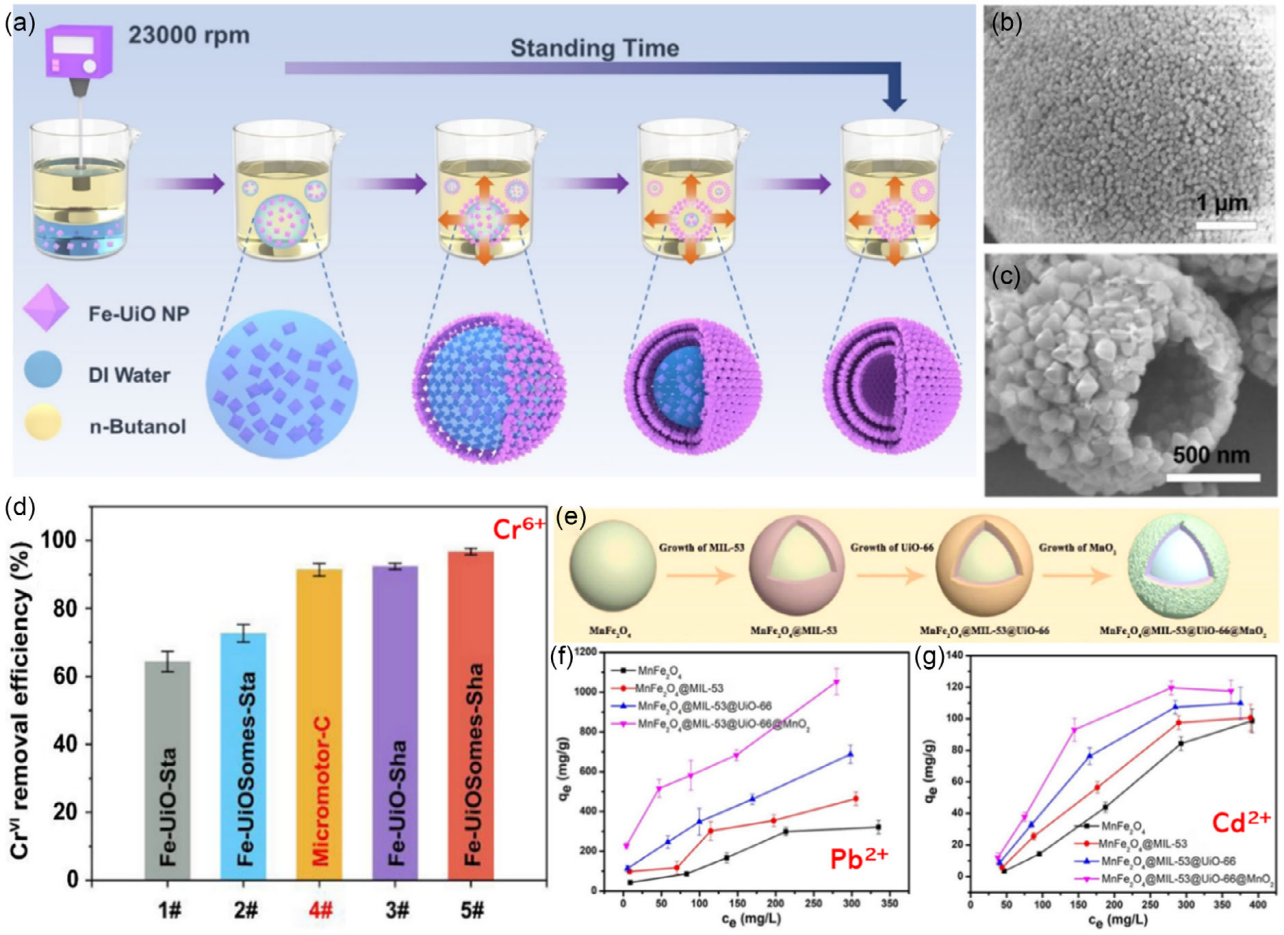
a) The fabrication process and Fe‐UiOSomes through the transient pickering emulsion method, b,c) high‐magnification SEM images of nonbroken and broken Fe‐UiOSomes, and d) adsorption of Cr^6+^ by different systems. Adapted with permission.^[^ ^125^ ^]^ Copyright 2022, John Wiley and Sons. e) Fabrication of the motor MnFe_2_O_4_@MIL‐53@UiO‐66@MnO_2_, f,g) The adsorption behaviors of Pb^2+^ and Cd^2+^ under different conditions. Adapted with permission.^[^ ^126^ ^]^ Copyright 2022, Elsevier.

The preparation of MOF‐MNMs was always tricky because of the difficulty of controlling the asymmetric structures. In addition, it was extensively reported that only microswimmers with a size larger than a critical size (almost 1 μm) would possibly be able to be propelled by bubbles.^[^ ^44^, ^85^, ^124^ ^]^ Wang and his team proposed a general strategy to prepare MOF‐MNMs by assembling nanosized MOF particles into a multilayer structure, resulting in a bigger particle size.^[^ ^125^ ^]^ They synthesized a bubble propulsion micromotor of Fe_3_O_4_@NH_2_‐UiO‐66 colloidosomes (Fe‐UiOSomes), which were further decorated with Pt‐NPs (denoted, Fe‐UiOSomes‐Pt) through the transient pickering emulsion method for a simultaneous removal of two types of pollutants: potassium dichromate Cr^6+^ and methyl orange dye. The fabrication process and SEM images are shown in **Figure** **15**a–c. Pt NPs were deposited on the motor by two different approaches: physical sputtering, producing Micromotor‐P, and chemical reduction, giving Micromotor‐C. It was found that the chemical deposition method was more effective. The average recorded speeds of both motors, in 5 wt% H_2_O_2_ and 1 wt% SDS, were 450 ± 180 and 303 ± 93 μm s^−1^ for Micromotor‐C and Micromotor‐P, respectively. The adsorption efficiency of Cr^6+^ by Micromotor‐C was 91% within 2 h, higher than that of Fe‐UiO‐Sta and Fe‐UiOSomes‐Sta (Figure 15d). The removal mechanism was attributed to the electrostatic attraction between (Cr^6+^) and the motor, which was enhanced by the motion‐induced convection between them.

In 2022, Li and co‐workers designed a self‐propelled MOFs‐coupled composite motor, MnFe_2_O_4_@MIL‐53@UiO‐66@MnO_2_, for the efficient adsorptive removal of heavy metal ions (Pb^2+^ and Cd^2+^).^[^ ^126^ ^]^ The hierarchical structure of the motor (Figure 15e) offered a high surface area, effective content of active sites, and good metal ion diffusion to the surface of the MOF. The binding sites of two MOFs increased the adsorption of both Pb^2+^ and Cd^2+^ at 1018 and 440.8 mg g^−1^, respectively (Figure 15f,g). The removal mechanism was attributed to three main reasons: 1) electrostatic attraction forces between the negatively charged motor and the positively charged metal ions; 2) partial ion exchange between metals in the motor (Mn and Fe) and the metal ion pollutants (Pb^2+^ and Cd^2+^); and 3) chemical interaction between the metals and the functional groups of the motor including –COOH groups.

From the above‐mentioned MOF‐MNMs and their adsorption behaviors toward different metal ions, predominant adsorption mechanisms can be discerned. 1) Adsorption of metal ions occurs within the porous framework of the material. 2) Electrostatic interaction between metal ions and specific active sites (acid–basic sites of ZIF‐8 or the imidazole nitrogen constituents) in certain MOFs plays a major role. 3) Direct chemical bonding between metal ions and functional moieties such as carboxyl (—COOH) groups in the MOF promotes the chemical adsorption.

**Scheme 2:**
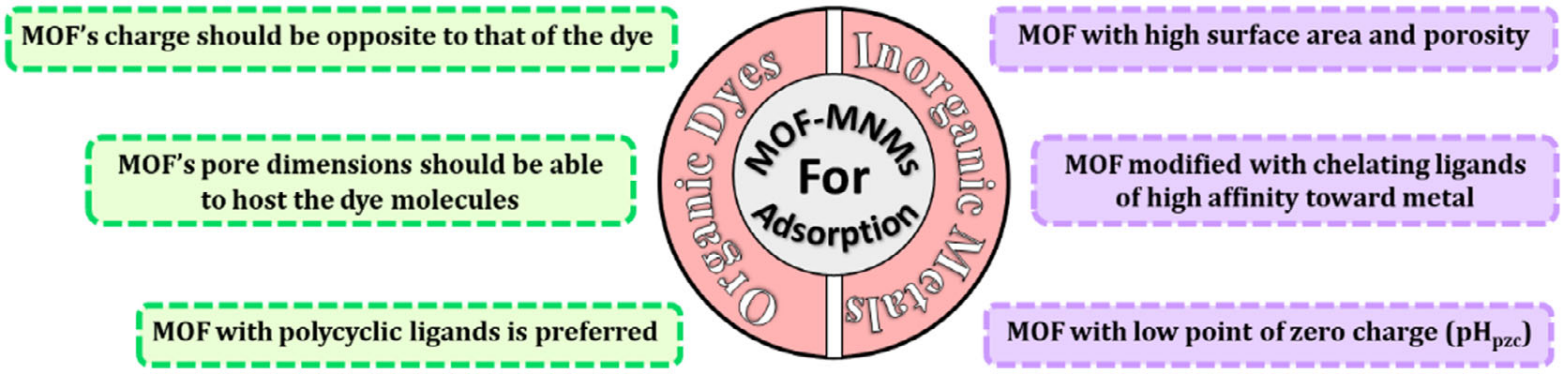
Criteria of good MOF‐MNMs for adsorption of metals and organic pollutants.

Thus, for enhanced removal efficacy, MOFs should ideally exhibit the following characteristics, as outlined in **Scheme** **2**. 1) It should possess a significant surface area and porosity to optimize metal ion adsorption within these cavities. 2) It should maintain a low point of zero charges (pH_pzc_) to maximize electrostatic interactions between the metal ions and the negatively charged MOF surface. 3) It should undergo functionalization with an appropriate chelating agent, such as ethylenediaminetetraacetic acid, in alignment with the hard–soft acid–base theory, ensuring optimal affinity to the targeted metal ions.

#### 5.1.2 Adsorption of Organic Dyes

Organic dyes are considered carcinogenic materials that can cause severe environmental problems to both human and aquatic life.^[^ ^127^ ^]^ Due to the widespread use of dyes in various industries, including textile, food, painting, and pharmaceutical products, the global annual consumption of dyes was estimated to be 7 × 10^5^ tons with a huge quantity, of almost 15% of the annual production of dyes, being discharged into water during synthesis and processing.^[^ ^128^ ^]^ Consequently, it is an urgent need to find a nonconventional method able to tackle this problem. Combining the autonomous motion of MNMs with the MOFs could enhance the contact with the dye pollutants, leading to higher depollution efficiencies.^[28a]^ Therefore, MOF‐MNMs could be a promising solution for such a problem and present the new era of adsorption technology.

**Figure 16:**
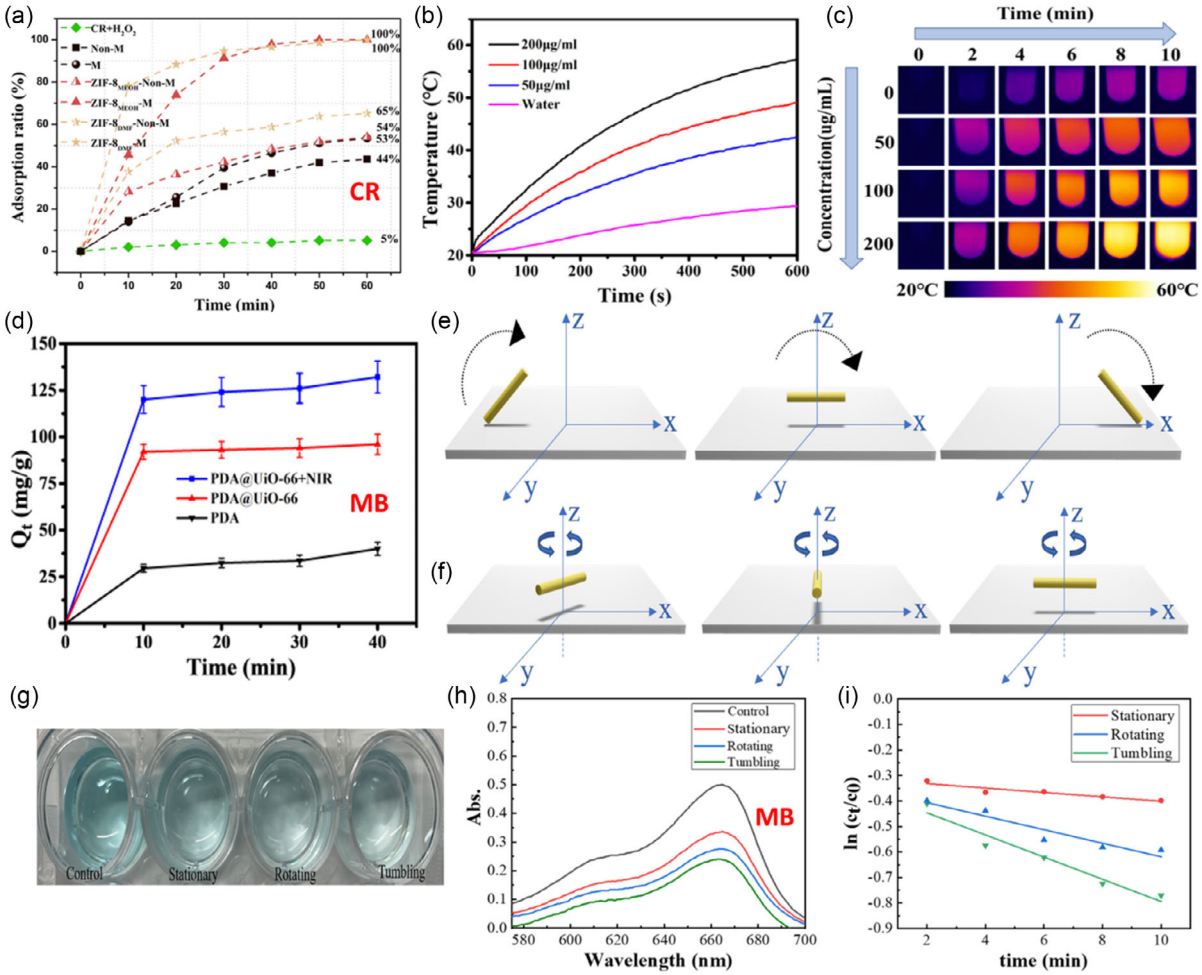
a) Adsorption ratio (%) of CR by ZIF‐8_MEOH_‐M and ZIF‐8_DMF_‐M. Adapted with permission.^[^ ^25^ ^]^ Copyright 2019, Elsevier. b,c) Temperature change profiles and the corresponding IR thermal images, respectively, of PDA@UiO‐66/water suspension with different concentrations of the motor (50, 100, and 200 μg mL^−1^) for 10 min, and d) adsorption capacities of MB under different conditions. Adapted with permission.^[^ ^27^ ^]^ Copyright 2022, Elsevier. e,f) Schematic diagrams of the model of the Fe_3_O_4_@Ce‐MOF tumbling and rotating at different time, respectively, g) optical image, h) UV–vis absorption spectra, and i) adsorption kinetics of MB after 10 min in the presence of the motor in different propulsion modes. Adapted with permission.^[28a]^ Copyright 2022, Elsevier.

Li and co‐workers reported a bioinspired ZIF‐8@γ‐Fe_2_O_3_/γ‐Al_2_O_3_/MnO_2_ magnetic tubular micromotor for the adsorption of different organic pollutants.^[^ ^25^ ^]^ They used the kapok fiber as a tubular template to build the micromotor on its inner and outer surfaces and prepared ZIF‐8_MEOH_‐M and ZIF‐8_DMF_‐M using methanol (MEOH) and N,N’‐dimethyformamide (DMF) as solvents, respectively. It was found that the speeds of ZIF‐8_MEOH_‐M and ZIF‐8_DMF_‐M in 1, 3, and 5% H_2_O_2_ were ≈30, 66, 150 and 38, 50, 105 μm s^−1^, respectively. The adsorption capacities (**Figure** **16**a) of ZIF‐8_MEOH_‐M and ZIF‐8_DMF_‐M for congo red (CR) were 309, and 394 mg g^−1^, respectively. The adsorption mechanism of the anionic CR molecules was attributed to the strong electrostatic interaction with the cationic zinc sites on the surface of the MOF, in addition to the *π*–*π* stacking interaction between CR aromatic rings and imidazole rings. Another motor, utilizing ZIF‐67, was studied toward removing methyl blue dye.^[^ ^116^ ^]^ ZIF‐67, with the spherical and dodecahedral morphologies, was prepared and spherical ZIF‐67 motor showed better adsorption performance than the dodecahedral structure, likely due to its higher porosity and surface area.

In 2022, Luan and his co‐workers fabricated a near‐infrared (NIR) light‐driven nanomotor of UiO‐66 deposited on mesoporous polydopamine (PDA) (abbreviated PDA@UiO‐66).^[^ ^27^ ^]^ IR radiation caused an asymmetric temperature gradient around the motor, accompanied by an overall temperature increase (Figure 16b), resulting in self‐thermophoretic propulsion with a maximum speed of 18.8 μm s^−1^ at 3 W cm^−2^ irradiation power density. The IR thermal images of PDA@UiO‐66/water suspension with different concentrations under (NIR) irradiation of 980 nm for 10 min are illustrated in Figure 16c. The motor was applied to remove methylene blue (MB) dye from water. PDA nanobowl, PDA@UiO‐66 (no light), and PDA@UiO‐66 (with NIR irradiation) presented the maximum adsorption capacities of 38, 89, and 134 mg g^−1^, respectively (Figure 16d). PDA@UiO‐66 offered more accessible active sites for adsorbing MB through the *π*–*π* interaction between the aromatic rings of MB and benzene rings of the UiO‐66 terephthalic acid.

A magnetically driven rod‐like Fe_3_O_4_@Ce‐MOF nanomotor was also used for MB removal.^[28a]^ The motor showed self‐propulsion under an externally applied magnetic field with two motion modes: tumbling motion and in situ rotation (Figure 16e,f), depending on the applied magnetic field settings. At 5 Hz, both the forward and the rotation speeds of the motor were 13.83 ± 3.05 μm s^−1^ and 296 ± 24 rpm, respectively, which would be increased to 16.69 ± 1.10 μm s^−1^ and 498 ± 113.6 rpm, respectively, at 10 Hz magnetic field. Both speeds started to decrease after 10 Hz, which is identified as step‐out frequency. The adsorption performance of the motor was examined in three motion modes: stationary, rotating, and tumbling motion (Figure 16g–i). The motor with a tumbling motion showed the highest adsorption efficiency and adsorption rate. In contrast, the static motor showed the lowest adsorption performance under the same magnetic field frequency of 10 Hz. Because its 3D molecular structure is smaller than the pore size of the motor, MB could be easily entrapped within the motor's pores.

**Figure 17:**
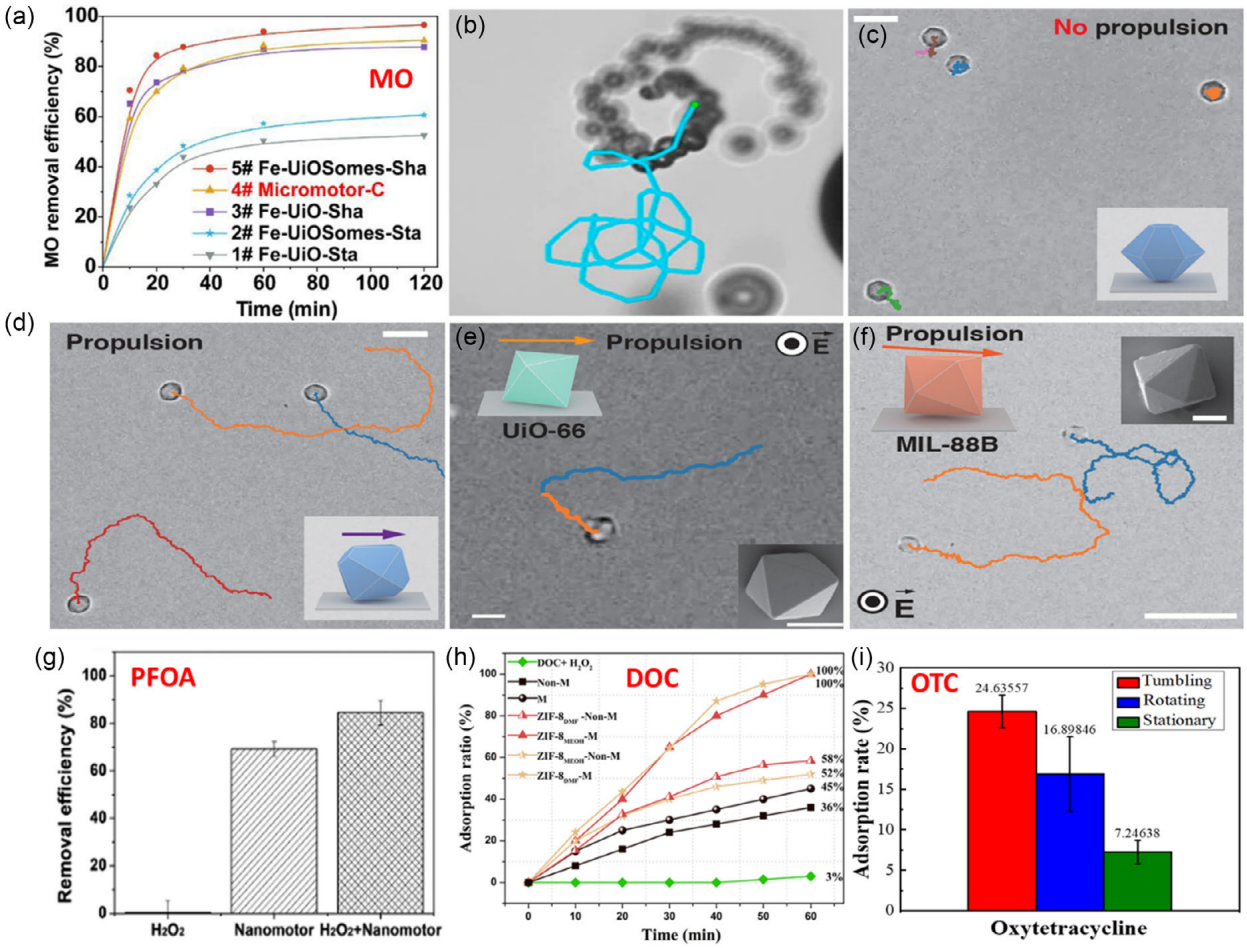
a) Adsorption of MO by Fe_3_O_4_@NH_2_‐UiO‐66‐Pt. Adapted with permission.^[^ ^125^ ^]^ Copyright 2022, John Wiley and Sons. b) The propulsion mode of UiO‐type Zr‐MOF in the presence of H_2_O_2_ and Triton X‐100 surfactant, respectively. Adapted with permission.^[^ ^118^ ^]^ Copyright 2020, American Chemical Society. c) Propulsion trajectories of MIL‐96 in the absence of AC, motion behavior of d) MIL‐96, e) UiO‐66, and f) MIL‐88B in the presence of electric field. Adapted with permission.^[^ ^29^ ^]^ Copyright 2021, American Chemical Society. g) Adsorption of PFOA by Cat‐ZIF‐8 motor. Adapted with permission.^[^ ^117^ ^]^ Copyright 2020, The Royal Society of Chemistry. h) Adsorption ratio (%) of DOC studied by ZIF‐8@γ‐Fe_2_O_3_/γ‐Al_2_O_3_/MnO_2_. Adapted with permission.^[^ ^25^ ^]^ Copyright 2019, Elsevier. i) Adsorption behavior of OTC by Fe_3_O_4_@Ce‐MOF motor. Adapted with permission.^[28a]^ Copyright 2022, Elsevier.

A Fe_3_O_4_@NH_2_‐UiO‐66‐Pt (denoted, Fe‐UiOSomes‐Pt) motor was tested to remove methyl orange (MO) (**Figure** **17**a).^[^ ^125^ ^]^ The adsorption mechanism of MO over the motor was mainly attributed to the electrostatic attraction between the positively charged surface of the motor and the negatively charged dye molecules in addition to the motion‐induced convection between them.

Sanchez et al. also fabricated a micromotor from a highly stable, presynthesized microporous UiO‐type Zr‐MOF.^[^ ^118^ ^]^ The motor showed a remarkable propulsion capability in the presence of 0.5% hydrogen peroxide fuel and Triton X‐100 surfactant (Figure 17b). The motor speed depended on the encapsulated catalase amount and the incubation time between MOF and the catalase enzyme. The maximum speed was 3.56 ± 0.56 body length/s and propelled for 7.0 ± 0.4 min. The motor was tested against RhB dye removal. The removal percentage was 51.0 ± 2.7% compared to only 14.6 ± 6.4% for the static motor (in the absence of both H_2_O_2_ and Triton X‐100), confirming the self‐propulsion positive effect on the adsorption process. The removal of RhB was attributed to the adsorption of the dye molecules onto the unoccupied micro‐ and mesoporous sites within the motor.

To sum up, MOF‐MNMs could function as adsorbents for different types of dyes through three main mechanisms: 1) electrostatic attraction forces between the dye molecules and the motor surface; 2) entrapping of the dye inside the pores of the MOF‐MNMs; and 3) *π*–*π* stacking interaction between the aromatic rings of the dye and the benzene rings of the ligands.

Therefore, designing a good MOF‐MNM as a dye adsorbent generally requires that the MOF chosen should have 1) an overall charge opposite to that of the dye for a good electrostatic attraction; 2) pore size and the pore dimensions to host the dye molecules; and 3) polycyclic ligands might enhance the adsorption capacities toward dyes through the *π*–*π* interaction (Scheme 2).

#### 5.1.3 Adsorption of Other Pollutants

MOF‐MNMs can move by O_2_ bubble, magnetic field, and light energy, and they can also move under an electric field as an external energy source. Wang et al. introduced a series of polyhedral micromotors of the MOFs (MIL‐96, UiO‐66, MIL‐88B, and ZIF‐8, denoted as M_1_, M_2_, M_3_, and M_4_, respectively), which can be electrically propelled.^[^ ^29^ ^]^ Under the effect of an AC (alternating current) electric field, the symmetry was broken, leading to an unbalanced electrohydrodynamic flow and propulsion. The four motors had different orientations, propulsion trajectories, and speeds under the electric field (Figure 17c–f). The maximum speeds of *M*_1_, *M*_2_, and *M*_3_ were 11.4, 4.27 ± 0.43, and 2.94 ± 0.24 μm s^−1^, respectively. In contrast, *M*_4_ did not show any propulsion because of its high symmetry. *M*_1_ was tested to remove fluoride (F^−^) ions, showing that 1 mg of the motor, under an AC electric field, could remove 0.004 mg of F^−^ within 30 min, which was 18% higher in the adsorption capacity than the nonpropelled motor. The adsorption was attributed to the motion‐induced mass transport of F^−^ within the channels and cavities of the MOF micromotor.

Perfluorooctanoic acid (PFOA) is widely used in industry and has been found in large quantities in water, soil, and seafood with a reported acute toxicity toward humans and animals.^[^ ^129^ ^]^ A Cat‐ZIF‐8 nanomotor was used to remove PFOA from water, and the removal efficiency was found to be 91.0% within only 2 min in 0.2% H_2_O_2_, compared to only 69% removal for the nonmobile motor, as illustrated in Figure 17g.^[^ ^117^ ^]^ The promising PFOA removal percentage was assigned to the adsorption on the pores of ZIF‐8, which is accompanied by strong adsorption energy.

During the COVID‐19 pandemic, antibiotics were extensively used, resulting in a considerable amount being drained into the water. MOF‐MNMs were used as adsorbents and applied to remove doxycycline (DOC) and oxytetracycline (OTC) from water. The bioinspired ZIF‐8@γ‐Fe_2_O_3_/γ‐Al_2_O_3_/MnO_2_ motor was used as a novel adsorbent for DOC, showing the maximum adsorption capacities of 119, 213, and 242 mg g^−1^ on M, ZIF‐8_MEOH_‐M, and ZIF‐8_DMF_‐M, respectively (Figure 17h).^[^ ^25^ ^]^ The adsorption mechanism of DOC was due to the *π*–*π* stacking interaction between the aromatic rings of both DOC and that of the organic linker.

A magnetic Fe_3_O_4_@Ce‐MOF motor was studied to remove OTC and the adsorption efficiency depends on the motion mode, which is highly dependent on the magnetic field settings.^[28a]^ Figure 17i shows that the adsorption of OTC in the case of tumbling mode is higher than that of the other two modes, where the adsorption rate in the tumbling was 24.6% compared to 16.9% and 7.2% in rotating and stationary mode, respectively. The molecular dimensions of OTC are 1.26 nm × 0.82 nm × 0.53 nm, which can be easily entrapped inside the pores of the motor.

### 5.2 MOF‐MNMs for Degradation

**Figure 18:**
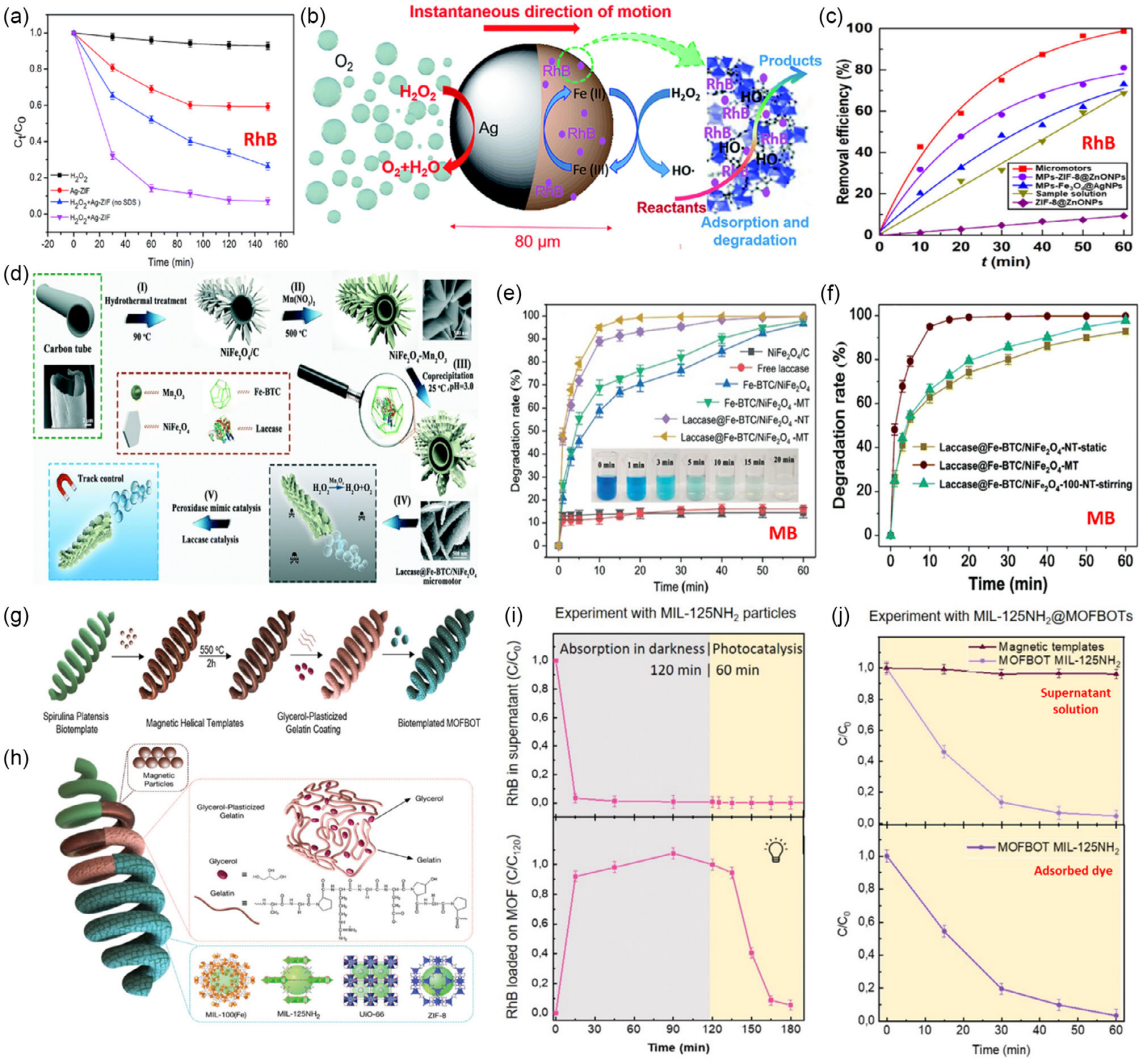
a) Removal behavior of RhB and b) propulsion and removal mechanisms by Ag‐ZIF micromotor. Reproduced with permission.^[^ ^119^ ^]^ Copyright, Royal Society of Chemistry. c) Photocatalytic degradation of RhB by ZIF8@ZnONPs|Fe_3_O_4_@AgNPs. Adapted with permission.^[^ ^130^ ^]^ Copyright 2020, American Chemical Society. d) Schematic illustration of the preparation and application of laccase@Fe‐BTC/NiFe_2_O_4_‐MT, e) removal of MB by different systems (the inset shows the MB color change during the degradation), and f) MB degradation by laccase@Fe‐BTC/NiFe_2_O_4_‐NT‐static (without mechanical stirring), laccase@Fe‐BTC/NiFe_2_O_4_‐MT and laccase@Fe‐BTC/NiFe_2_O_4_‐100‐NT‐stirring (mechanical stirring with a speed of 100 rpm). Adapted with permission.^[^ ^131^ ^]^ Copyright 2020, The Royal Society of Chemistry. g) The fabrication process of the biotemplated MOFBOTs, h) a schematic illustration of the MOFBOTs showing its all components, and i) removal of RhB by MIL‐125NH_2_ particles via adsorption and photocatalysis, and j) declining of RhB concentration from the supernatant solution (top) and adsorbed on the motor (bottom) by 125NH_2_(Ti)@MOFBOTs during the photocatalysis. Reprinted with permission.^[28b]^ Copyright 2021, John Wiley and Sons.

Besides its extensive usage to remove different contaminants by adsorption, MOF‐MNMs have been reported widely for oxidative degradation of water pollutants. Guo et al. designed a self‐propelled polystyrene@ZIF‐Zn‐Fe/Ag (Ag‐ZIF) micromotor to degrade RhB dye.^[^ ^119^ ^]^ As illustrated in **Figure** **18**a, the removal reached 47% on the motor without H_2_O_2_, which might be attributed to surface adsorption. In contrast, a degradation rate of 93.1% was observed by the motor in 12% H_2_O_2_ within 150 min. Besides the adsorption on the surface of the motor, the removal mechanism of RhB was also attributed to oxidative degradation of the dye by the oxidizing hydroxy radicals (.OH), as illustrated in Figure 18b.

Wang and co‐workers reported a porous asymmetric spherical motor (ZIF‐8@ZnONPs|Fe_3_O_4_@AgNPs) for photocatalytic degradation of RhB.^[^ ^130^ ^]^ The effective removal of RhB at 98.6% could be achieved in the presence of H_2_O_2_ and UV irradiation within 60 min (Figure 18c). The removal mechanism was attributed to the adsorption of pollutant molecules, within the porous surface of MOF, and photocatalysis.

Other motors based on the bubble propulsion mode and the synergistic removal mechanism were used for the degradation of MB. Wang et al. reported that Fe_3_O_4_/ZIF‐67(Co) micromotor, propelled by the asymmetric decomposition of H_2_O_2_ with a speed of 115 μm s^−1^ at 10% H_2_O_2_, achieved high degradation efficiency of MB (90%) within only 1 h.^[^ ^121^ ^]^ Besides its adsorption onto the porous surface of the motor, MB was degraded by the synergistic Fenton oxidation caused by both Fe^2+^ and Co^2+^.

A laccase@Fe‐BTC/NiFe_2_O_4_‐Mn_2_O_3_ tubular micromotor, laccase@Fe‐BTC/NiFe_2_O_4_‐MT (Figure 18d), was fabricated by Li and co‐workers.^[^ ^131^ ^]^ The motor can move in a circular‐like pattern by the oxygen thrust formed from the asymmetric catalytic decomposition of hydrogen peroxide over Mn_2_O_3_, with a speed of 300 ± 27.5 μm s^−1^ in 5% H_2_O_2_. Figure 18e illustrates the degradation of MB in the presence of different systems. Laccase@Fe‐BTC/NiFe_2_O_4_‐MT could degrade 100% of the dye within only 20 min compared to 98, 14, and 15% for Fe‐BTC/NiFe_2_O_4_‐MT, NiFe_2_O_4_, and free laccase, respectively. The removal mechanism was proposed based on the synergistic effect of Fenton, peroxidase, and laccase catalytic activities. As shown in Figure 18f, Laccase@Fe‐BTC/NiFe_2_O_4_‐MT exhibited a higher degradation activity than both static laccase@Fe‐BTC/NiFe_2_O_4_ and laccase@Fe‐BTC/NiFe_2_O_4_‐NT (nonmicromotor without Mn_2_O_3_, under mechanical stirring with a speed of 100 rpm), indicating the positive contribution of autonomous movement. In addition, the mechanism was proposed based on the synergistic catalytic effect of Fenton, natural laccase enzyme, and the nanozymes (Fe‐BTC/NiFe_2_O_4_).

Motors with dual functions have attracted significant attention in water‐related applications. For example, MOF‐MNMs were used for the degradation and detection of hydroquinone^[^ ^110^, ^132^ ^]^ and the degradation of RhB and doxorubicin (DOX) drug delivery.^[28b]^ Luis et al. synthesized a series of biotemplated MOF‐based small‐scale robots (MOFBOTs) using *S. platensis* cyanobacteria as a biotemplate (Figure 18g,h).^[28b]^ Four different MOFs were used including MIL‐100(Fe), MIL‐125NH_2_(Ti), UiO‐66(Zr), and ZIF‐8(Zn) giving MIL‐100(Fe)@MOFBOTs, MIL‐125NH_2_@MOFBOTs, UiO‐66@MOFBOTs, and ZIF‐8@MOFBOTs, respectively. The magnetic properties of the motors made it a good candidate to be powered by an external magnetic field. The speed and propulsion scheme of the motors strongly depended on the applied frequency. The motors showed a wobbling propulsion scheme between 1 and 5 Hz, whereas the motion became corkscrew between 6 and 16 Hz. The maximum velocity was almost 28 μm s^−1^ at 17 Hz for MIL‐100(Fe)@MOFBOTs.

Then MIL‐125NH_2_(Ti)@MOFBOTs was tested for photocatalytic degradation of RhB under UV–vis light irradiation. For the removal of RhB by MIL‐125NH_2_ particles (Figure 18i), the concentration of RhB decreased with time before the photocatalytic degradation process, attributed to the adsorption on the porous structure. Once the irradiation started, the adsorbed molecules started to decrease due to the degradation process, reaching more than 90% degradation within 60 min. Figure 18j shows the decreasing RhB concentration either from solution or those adsorbed on the motor. The results showed that both RhB in solution and adsorbed on the motor were degraded by the active hydroxy radicals (^•^OH) formed during the photocatalysis.

**Scheme 3:**
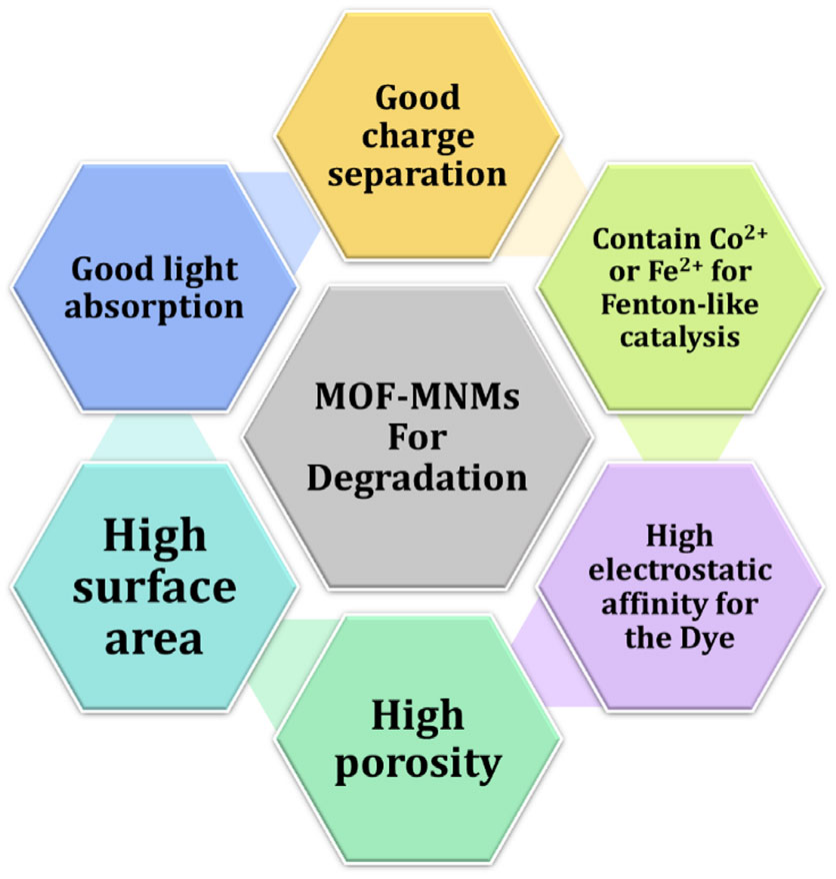
Conditions for highly effective MOF‐MNMs for pollutant degradations.

To summarize, MOF‐MNMs work via two main steps: first, adsorption of the pollutant molecules on the motor surface, and then the oxidative degradation process, which might be either photocatalytic degradation or Fenton oxidation. So, for the MOF‐MNM to be highly effective in degradation, as illustrated in **Scheme** **3**, it is better to: 1) have both a high adsorption ability (high surface area, high porosity, and high electrostatic affinity toward the dye molecules) and 2) have high catalytic activities (like good semiconductor properties, good e‐h charge separation, and for the Fenton‐like oxidation, it must have either iron or cobalt or both inside the MOF structure).

### 5.3 MOF‐MNMs for Sensing

Although MOF has been extensively used for sensing different types of pollutants, including toxic metals, chemical‐, and biological‐related materials,^[^ ^133^ ^]^ we have found only a few articles on MOF‐MNM for detecting various substances. It was found that the MOF‐based motors for sensing can work through two main mechanisms: fluorescence quenching and colorimetric detection.

#### 5.3.1 Fluorometric Sensing

**Figure 19:**
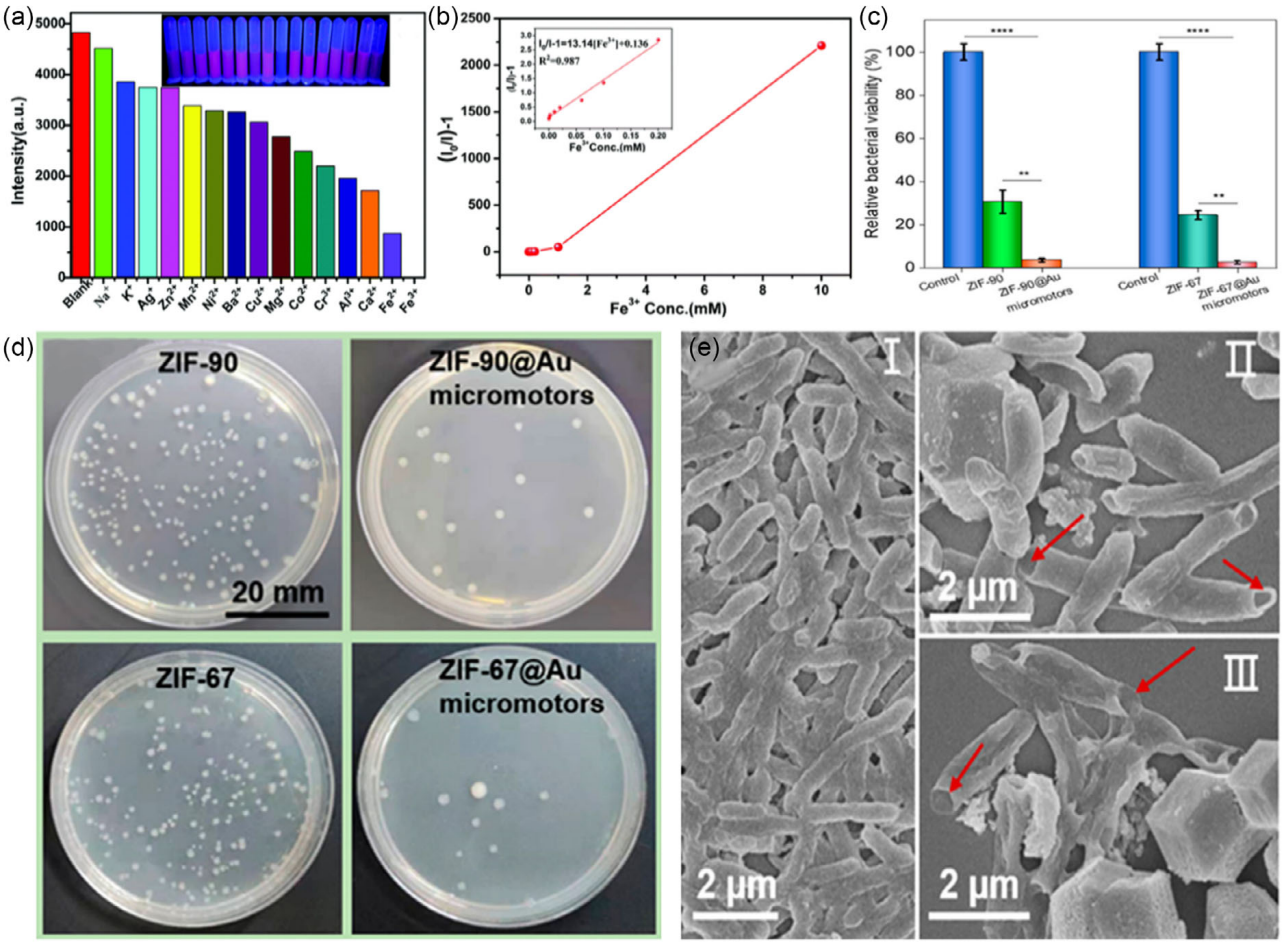
a) Fluorescence response of Eu‐MOF/EDTA‐NiAl‐CLDH/MnO_2_ toward different metal ions (the inset shows photographs taken under UV light) and b) stern‐Volmer plot for the fluorescence quenching by Fe^3+^ (the inset shows the linear range). Adapted with permission.^[^ ^109^ ^]^ Copyright 2019, The Royal Society of Chemistry. c) The bacterial viabilities and d) photographs of bacterial colonies of *E. coli* after treatment with different materials, and e) SEM images of *E. coli* cells after different treatments: (I) H_2_O, (II) ZIF‐90@Au, and (III) ZIF‐67@Au micromotors. Adapted with permission.^[^ ^30^ ^]^ Copyright 2022, American Chemical Society.

Li and co‐workers engineered a fluorescent micromotor composed of Eu‐MOF/EDTA‐NiAl‐CLDH/MnO_2_ for removing and detecting Fe^3+^ ions in water.^[^ ^109^ ^]^ The motor showed a strong fluorescence emission at 618 nm upon excitation with 306 nm light. **Figure** **19**a shows the fluorescence emission intensity of the motor before and after adding different metal ions (Na^+^, K^+^, Ag^+^, Zn^2+^, Mn^2+^, Ni^2+^, Ba^2+^, Cu^2+^, Mg^2+^, Co^2+^, Cr^3+^, Al^3+^, Ca^2+^, Fe^2+^, and Fe^3+^). Upon adding Fe^3+^, a total fluorescence quenching occurred for the motor, suggesting a very high specificity and sensitivity of the motor toward Fe^3+^ ions. A linear range at 0–0.2 mm and a limit of detection (LOD) of 0.15 μm were obtained in Figure 19b. The fluorescence quenching of the motor by Fe^3+^ ions was explained by 1) the competition between Fe^3+^ and the motor (Eu‐MOF/EDTA‐NiAl‐CLDH/MnO_2_) to absorb the same excitation wavelength due to the overlap in their absorption spectra and 2) the possible electron transfer from the excited state of Eu‐MOF to the half‐filled 3*d* orbital of Fe^3+^.

#### 5.3.2 Colorimetric Sensing

Due to the extensive use of hydroquinone (HQ) in different areas of industry, including cosmetics, pesticides, and pharmaceutical products, it is widely drained into water, which might cause severe problems for aquatic life and human health.^[^ ^134^ ^]^ Li et al. developed a magnetic micromotor using horseradish peroxidase‐MIL‐100(Fe)‐Mn_2_O_3_@TiO_2_@Fe_3_O_4_ (HRP‐MIL@TiO_2_‐MMT) for efficient colorimetric detection of HQ.^[^ ^110^ ^]^ The speed was 48.3 ± 4.3 μm s^−1^ in 0.5% H_2_O_2_, which increased to 786 ± 37.5 μm s^−1^ in 7% H_2_O_2_. The colorimetric detection strategy relies on using a suitable chromogenic substrate that forms a specific color or changes its color upon reacting with the analyte. In this context, 3,3’,5,5’‐tetramethylbenzidine (TMB) was used as a colorimetric probe for detecting HQ. In a typical detection experiment, TMB is oxidized to deep blue ox‐TMB in the presence of H_2_O_2_ and HRP‐MIL@TiO_2_‐MMT motor. In the presence of HQ, which possesses a strong reduction power, the ox‐TMB is highly reduced back to TMB, resulting in a noticeable color change from blue to colorless. HRP‐MIL@TiO_2_‐MMT was found to be effective for HQ detection within the range of 2–240 μm, achieving LOD at 1.84 μm.

Another flower‐like magnetic micromotor, Fe_3_O_4_@MnO_2_@HKUST‐1, has also been reported to detect HQ using the same colorimetric detection mechanism.^[^ ^132^ ^]^ The motor showed a maximum velocity of 99 ± 2 μm s^−1^ in 5% H_2_O_2_ with a linear concentration range of 1–280 μm and LOD of 0.94 μm.

Currently, MOF‐MNM sensors generally work through two different pathways: luminescence quenching by different pollutants and colorimetric sensing using specific chromogenic agents.

**Scheme 4:**
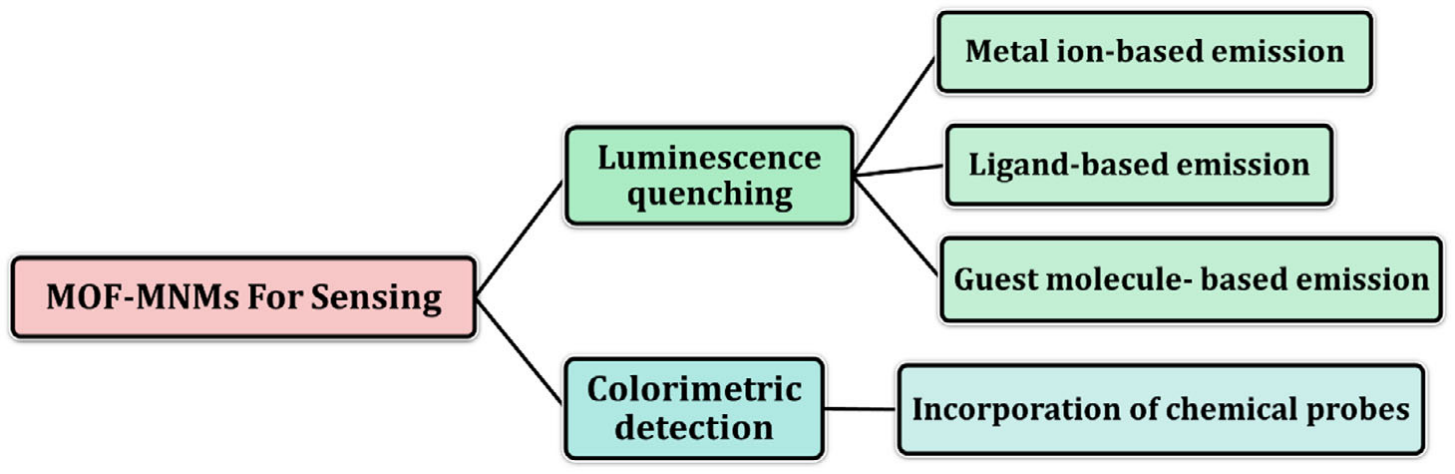
Different strategies to enhance MOF‐MNMs for sensing applications.

To expand the use of MOF‐MNMs in sensing applications, we strongly recommend the following consideration (**Scheme** **4**). 1) Expand the strategies of generating emissions from the MOF through different approaches like metal ion‐based emission, organic ligands‐based emission, and guest molecules‐based emission, as described elsewhere,^[^ ^135^ ^]^ rather than depending only on the Eu‐based MOFs. 2) Expand the colorimetric detection by incorporating chemical probes inside the MOF structure instead of only using external chromogenic agents, as mentioned elsewhere.^[^ ^136^ ^]^

### 5.4 MOF‐MNMs for Water Disinfection

Microorganisms, including bacteria, are another class of water pollutants causing harmful effects on human health. Because conventional methods cannot effectively remove microorganisms in a largescale, a nonconventional solution for such a problem is required. Yan and co‐workers reported Janus MOF@Au MNMs with unique self‐antibacterial activities.^[^ ^30^ ^]^ Different MOFs (ZIF‐90, ZIF‐67, ZIF‐8, MOF‐5, and UiO‐66) were water sensitive and released their metal ions in water. However, the Au layer prevents the release of ions from the coated side of the motor. The released metal ions on the uncoated side would power the micromotors by ionic self‐diffusiophoresis in the direction of the coated side. The speeds of ZIF‐90@Au, ZIF‐67@Au, ZIF‐8@Au, and ZIF‐5@Au were ≈15.9, 17.2, 9.7, and 5.6 μm s^−1^, respectively, while UiO‐66 showed no self‐propulsion in water and only performed a Brownian motion.

ZIF‐90@Au and ZIF‐67@Au were used to test their antibacterial activities against *Escherichia coli* (*E. coli*) through plate counting protocol. In a typical disinfection experiment, equivalent amounts of ZIF‐90, ZIF‐90@Au, ZIF‐67, and ZIF‐67@Au were separately dispersed in 2 mL of bacterial solution and incubated for 30 min. Then, the solid materials were removed, and the bacterial suspension was incubated overnight, followed by counting the number of colonies formed. It was found that, the bacterial death rates of *E. coli* by ZIF‐90@Au and ZIF‐67@Au (Figure 19c,d) were 96.3% and 97.4%, respectively, which were relatively higher than those of ZIF‐90 (69.3%) and ZIF‐67 (75.4%). The motor motion could improve the contact with the bacterial cells for enhanced antibacterial activities. The SEM images of *E. coli* (Figure 19eII,III) showed vigorous damage with many pores on the cell membrane. The mechanism of antibacterial activity of the motors can be well‐explained through two steps: the micromotors of ZIF‐90@Au and ZIF‐67@Au released their metal ions Zn^2+^ and Co^2+^, respectively, and the released metal ions disrupted the bacteria cell membrane due to a strong electrostatic interaction with its wall, leading to the leakage of cellular components and loss of cell metabolism, ultimately resulting in bacterial death.

Several strategies have been introduced for water disinfection by MOF, via singlet‐oxygen generation from MOF^[^ ^137^ ^]^ and AgNPs‐decorated MOF.^[^ ^138^ ^]^ In addition, decorating MOF with singlet oxygen photosensitizers could offer a new paradigm for water disinfection.^[^ ^139^ ^]^ Therefore, these strategies can be further applied for MOF‐MNMs to obtain a good disinfection ability in addition to the great self‐propulsion capability.

## 6 Conclusions and Outlook

This review has extensively studied the different MOF‐MNMs and their water‐related applications, including adsorption, catalytic degradation, sensing of different types of pollutants, and water disinfection. Although MOFs are studied in different applications owing to their promising and outstanding properties, their implementation as micromotors requires more exploration. Currently, MOF‐MNMs are tested for the adsorption of different pollutants, including metals, dyes, anions, antibiotics, and other organic pollutants.

The motion of MOF‐MNMs is generally powered by different energy sources, including bubble propulsion, light, NIR irradiation, magnetic field, and electric field. In addition, MOF‐MNMs can be propelled in the absence of any chemical fuel or external physical stimulus. Among the different energy sources used for MOF‐MNMs, hydrogen peroxide was one of the most studied fuels. Different catalysts were used in MOF‐MNMs for H_2_O_2_ decomposition, including MNPs, metal oxides (MnO_2_, Mn_2_O_3_, Fe_3_O_4_), and enzymes. It was found that the highest speed was obtained using MNPs (AgNPs and PtNPs), but it usually requires a high fuel concentration, in some cases at 20–30%. In contrast, the enzymes are the most effective catalysts that can propel the motor even at a very low concentration of fuel, for example, 0.2%.

The physically driven MOF‐MNMs have been also covered through the light, magnetic field, and electric field as a driving power. Although their speeds are relatively low compared to those of the chemically powered MOF‐MNMs, they are not only much safer but also showed better removal of different contaminants compared to the nonpropelled MOFs. In addition to the chemically powered and the physically driven MOF‐MNMs, some MOFs exhibited an intrinsic self‐propulsion capability in water, which is considered as one more merit of MOFs.

For the adsorption of metal ions, MOF‐MNMs should have a high surface area and porosity, low pH_pzc_, and functionalized with ligands in high affinity toward the metal ions of interest.

For degradation, the MOF‐MNMs should possess an overall charge opposite to the charge of the pollutants with a pore size to host the pollutants. Furthermore, the degradation usually occurs through two main steps: adsorption and oxidative degradation. Therefore, a highly effective MOF‐MNM for degradation must have a sensitive component for oxidant (H_2_O_2_) activation and energy conversion to induce oxidation reaction.

The detection process of different kinds of pollutants (metals and organic compounds) has also been covered via both fluorescence quenching and colorimetric detection. Chemical sensing by MOF‐MNMs needs more research attention. We can focus on the MOFs with metal ion‐based emission, organic ligands‐based emission, and guest molecules‐based emission as well as incorporating different chemical probes inside the MOF structure for different sensing purposes.

Moreover, MOF‐MNMs can be used for bacterial disinfection. However, the current MOF‐MNMs in water disinfection depended on motor disintegration. Different approaches can effectively broaden the application of water disinfection by MOF‐MNMs to be used for more harmful microorganisms, like viruses. MOFs with singlet‐oxygen generation ability and AgNPs decorated MOF can form good MOF‐MNMs for water disinfection.

Although the MOF‐MNMs in water remediation cover almost all types of applications, it needs more effort from the researchers to create novel and more effective MOF‐MNMs able to remove different kinds of pollutants.

## Conflict of Interest

The authors declare no conflict of interest.
